# Deciphering the impact of *SNAI1* gene on renal tubular cell proteome, nucleolar stress, ribosome biogenesis, senescence, DNA damage response, and focal adhesion dynamics

**DOI:** 10.1016/j.gendis.2025.101926

**Published:** 2025-11-10

**Authors:** Rattiyaporn Kanlaya, Kanokwan Nonthawong, Mueanchan Suntivichaya, Sunisa Yoodee, Visith Thongboonkerd

**Affiliations:** Medical Proteomics Unit, Research Department, Faculty of Medicine, Siriraj Hospital, Mahidol University, Bangkok 10700, Thailand

**Keywords:** Epithelial–mesenchymal transition, Kidney, Proteomics, Renal fibrosis, Senescence, *SNAI1*

## Abstract

Snail1, encoded by *SNAI1* gene, is an essential protein that regulates epithelial–mesenchymal transition, which leads to extracellular matrix accumulation and kidney fibrosis, but with unclear cellular and molecular mechanisms. This study compared the cellular proteome of *SNAI1*-overexpressed renal tubular cells with that of vector-control cells by label-free quantitative proteomics, followed by functional assessments using various assays. A total of 233 proteins showed significant changes in their levels by ectopic *SNAI1* expression. Of these, immunoblotting confirmed the decreases in HSP60 and HSP70 and the increase in DDX1. Bioinformatic analyses revealed the top 10 transcription factors as key upstream regulators of the altered cellular proteome, and translational regulation, ribosome, cell cycle regulation, and cellular senescence were primarily associated with these altered proteins. Gene ontology enrichment showed that focal adhesion, the structure where cells maintain their interior-extracellular matrix interactions, was one of the major affected cellular components. Experimental validations demonstrated that *SNAI1*-overexpressed cells displayed increases in nucleophosmin, nucleolar organizer regions, cell size, granularity, p21, γH2AX, MMP-9 secretion, and paxillin expression, confirming the bioinformatic predictions. This study has broadened our knowledge of Snail1 functions beyond its established role as the epithelial–mesenchymal transition regulator. In addition to alterations in the cellular proteome, ectopic *SNAI1* expression induced nucleolar stress, ribosome biogenesis, senescence, and DNA damage response in renal tubular cells. Moreover, Snail1 also affected the dynamics of focal adhesion, which is imperative for cell migration, by regulating paxillin expression. These findings may offer new therapeutic targets related to Snail1-dependent mechanisms for effective management of kidney fibrosis.

## Introduction

Chronic kidney disease (CKD) is a substantial health burden worldwide, impacting millions of individuals globally.^1^ The disease prevalence has been increasing steadily, driven by rising rates of its associated diseases, especially diabetes, hypertension, and cardiovascular disorders.^1^^,^^2^ The main manifestation of CKD is kidney fibrosis, a progressive pathological condition featured by excessive buildup of extracellular matrix (ECM) components that disrupt normal kidney architecture and functions, irrespective of the initial insult.^3^ Kidney fibrosis is driven by complex interplays of inflammatory mediators, oxidative stress, and cellular processes, particularly epithelial–mesenchymal transition (EMT) when tubular epithelial cells lose their epithelial appearances (*e.g.*, cell polarity and junctional complex) but obtain fibroblast-like mesenchymal phenotypes, including increased motility.^3^^,^^4^ While transient EMT facilitates effective tissue repair following injury, sustained EMT can contribute to fibrosis, tissue degeneration, and organ failure.^5^ These phenotypic changes are regulated by multiple transcription factors that modulate genes essential for EMT progression.^6^ Among these regulators, Snail1 acts as a master switch of EMT.^7^

Snail1 is a small transcription regulatory protein encoded by the *SNAI1* gene located on chromosome 20q13.2 that belongs to the Snail superfamily of zinc-finger transcription factors (“Snail1” refers to the protein, whereas “*SNAI1*” refers to its gene).^8^ This transcription factor is crucial for regulating other genes involved in EMT processes.^7^ Regulation of Snail1 involves multiple mechanisms, ranging from gene transcription to protein modifications, which determine its dual functions as a transcriptional repressor or activator based on interactions with diverse partners.^9^ Multiple signaling pathways can regulate Snail1 at different levels, including transcriptional activation, protein stability, and cellular localization.^10^, ^11^, ^12^, ^13^ Recently, the role of Snail1 in kidney fibrosis has been widely explored. Snail1 expression is often elevated in response to harmful stimuli, such as ischemia-reperfusion injury and chronic inflammation.^7^ Upon activation, Snail1 acts by repressing epithelial regulators while activating mesenchymal regulators, thereby facilitating the phenotypic transition of tubular epithelial cells into myofibroblasts, the major producers of ECM. This process leads to excessive accumulation of scar locales and impaired functions of the kidney.^7^

A study has demonstrated that Snail1 reactivation triggers partial EMT in injured tubular epithelial cells with downstream signaling at the interstitium to activate myofibroblast, fibrotic processes, and chronic inflammation, all of which are essential for the development of kidney fibrosis.^14^ Nevertheless, *in vivo* experiments have illustrated that Snail1-induced fibrosis can be restored. By targeting Snail1 expression, therapeutic interventions in mice with obstructive nephropathy can ameliorate kidney damage.^14^ In line with this, nodakenin from *Angelicae gigas* is effective for reducing tubulointerstitial fibrosis in unilateral ureteral obstruction mice by inhibiting Snail1 activity and curbing inflammation.^15^ Nodakenin also reduces Snail1 expression and Smad3 phosphorylation in transforming growth factor beta 1 (TGF-β1)-treated renal epithelial cells, suggesting that its therapeutic effects are likely mediated via Snail1-dependent pathways.^15^

Recent advances in proteomics have provided critical insights into the pathophysiology of CKD. For example, a large-scale study has linked the decline in kidney function to changes in the plasma proteome, highlighting tumour necrosis factor-related pathways in inflammatory and fibrotic processes.^16^ In a fibrosis-resistant spiny mouse model, proteomic analysis has identified decreases in pro-fibrotic ECM proteins, and cellular assays have shown the anti-fibrotic phenotypes in macrophages, providing an avenue for developing new CKD treatments.^17^ In an experimental model of CKD, integrated transcriptomic and proteomic analyses have identified dysregulated molecules primarily linked to fibrosis, metabolism, and immune response, with lumican and collagen alpha-1(III) chain as promising urinary biomarkers.^18^ Accordingly, urinary peptidomics has been proven valuable for monitoring kidney health with urinary peptide signatures that reflect collagen turnover in healthy and CKD states.^19^ Moreover, urinary proteomics of CKD patients has revealed some proteins as the aging-related biomarkers, which are well-correlated with age, kidney function, fibrotic markers, and glomerulosclerosis.^20^

Despite the mentioned knowledge on Snail1 roles in kidney fibrosis, the cellular and molecular mechanisms underlying its regulatory networks and signaling pathways remained unclear. To address this gap and to open avenues for targeted therapies and effective remedies to cease or reverse CKD progression, this study has established renal tubular cells with stably overexpressed Snail1 and compared their cellular proteome profile with that of vector-control cells by label-free quantitative proteomics. The differentially expressed proteins induced by ectopic *SNAI1* expression were subjected to functional enrichment analyses followed by experimental validations by various assays.

## Materials and methods

### Generation of *SNAI1*-overexprssed HK-2 cell line

A stable *SNAI1*-overexpressed HK-2 cell line was established by retroviral-mediated pBabe-puro-Snail transduction to enable stable integration of a gene of interest into the host cell genome, resulting in sustained gene expression. This retroviral system required plasmids as follows. The pBabe-puro-Snail plasmid (Addgene #23347; kindly provided by Bob Weinberg), which contained the human *SNAI1* gene encoding the transcription factor Snail1 along with a puromycin-resistance cassette, served as the transfer vector. The empty vector pBabe-puro (Addgene #1764; kindly provided by Hartmut Land), which lacked the *SNAI1* insert, was used as the transduction control. The pUMVC plasmid (Addgene #8449; kindly provided by Bob Weinberg) contained *gag* and *pol* genes required for retroviral core protein production and reverse transcription. The pCMV-VSV-G plasmid (Addgene #8454; kindly provided by Bob Weinberg) encoded the vesicular stomatitis virus G (VSV-G) envelope glycoprotein, which facilitated efficient viral entry into a wide range of mammalian cells.

Retroviral particles were produced in HEK 293T cells (ATCC) via transient co-transfection of a three-plasmid system. HEK 293T cells were seeded at 1 × 10^6^ cells per 100-mm dish and cultured overnight. The cells were then co-transfected with pBabe-puro-Snail (or empty vector), pUMVC, and pCMV-VSV-G using a lipofectamine transfection reagent according to the protocol provided by the manufacturer (Invitrogen). The culture medium was then refreshed at 16-h post-transfection. Following 48 h, the supernatant containing released viral particles was collected and filtered through a 0.45-μm syringe filter before being introduced to HK-2 cells (ATCC) (the target cells set at 40% confluence). The viral transduction was done at 37 °C for 3 h, followed by incubation in a fresh complete medium (10 mL/dish) for 48 h. The transduced HK-2 cells were selected in a selective medium containing 0.6 μg/mL puromycin for 7–10 days.

Following selection with puromycin, the clonal selection was performed to ensure the generation of a homogeneous cell population with consistent transgene integration and expression. Briefly, puromycin-resistant cells were seeded into a 96-well plate (one cell per well) to allow the formation of individual colonies derived from single cells. Once colonies became visible, individual clones were manually picked and expanded under continuous puromycin selection. Each clone was subsequently screened by Western blotting analysis to verify successful and stable overexpression, as indicated by elevated Snail1 protein level relative to the control cells transduced with viral particles containing the empty vector.

### In-solution tryptic digestion, nanoflow liquid chromatography-electrospray ionization-linear ion trap-Orbitrap (nanoLC-ESI-LTQ-Orbitrap) tandem mass spectrometry (MS/MS), and label-free quantitative proteomics

Whole cell lysate was prepared in SDT lysis buffer comprising 4% SDS, 100 mM DTT, and 100 mM Tris–HCl (pH 7.6). Upon determining protein concentrations, 30 μg of proteins per sample was processed by in-solution tryptic digestion according to the protocol previously described.^21^ The generated peptides were separated by nanoLC-ESI-LTQ-Orbitrap MS/MS as previously reported.^21^^,^^22^ The generated output format (.RAW) files were analyzed by MaxQuant (version 2.1.4.0) integrated with the Andromeda search engine. The following parameters were used for the identification of proteins within the UniProtKB/Swiss-Prot (human) database: carbamidomethylation at cysteine (C) as fixed modification; oxidation at methionine (M) as a variable modification; trypsin as the proteolytic enzyme; only one missed cleavage was allowed; precursor mass tolerance was 4.5 ppm; fragment mass tolerance was 0.5 Da; and charge state = +2, +3. A false discovery rate (FDR) at 1% cutoff was applied at both peptide-spectrum match (PSM) and protein levels. The MaxQuant LFQ (MaxLFQ) algorithm, featuring match-between-runs, was used for label-free protein quantification (LFQ). All other MaxQuant settings were maintained at their default values as described in the previous study.^23^

The obtained ProteinGroups.text was further analyzed by Perseus (version 2.0.7.0). The protein hits flagged as potential contaminants corresponding to decoy database entries and those identified only by site modifications were filtered out. Only proteins detected consistently across all replicates within each group were retained. The generated LFQ intensity was log2-transformed. Median normalization was applied to the data before mean difference analysis using the Student’s *t*-test. *P*-values <0.05 were considered statistically significant.

### Deciphering the biological significance of the altered cellular proteome

To predict the upstream regulatory networks relevant to the alterations in cellular proteome induced by *SNAI1* overexpression, substantially changed proteins were subjected to X2K Appyter (Expression2Kinases) (https://appyters.maayanlab.cloud/X2K_Appyter) enrichment analysis to obtain the top 10 transcription factors. The interacting network among the transcription factors together with Snail1 was visualized by the STRING (search tool for the retrieval of interacting genes/proteins) database (version 12.0) (https://string-db.org). Functional enrichment analyses using the STRING and Kyoto encyclopedia of genes and genomes (KEGG) pathway (https://www.genome.jp/kegg/pathway.html) databases were specifically performed on proteins regulated by the predicted transcription factors to obtain the biological significance induced by *SNAI1* overexpression. The connection between proteins and the different KEGG pathways was depicted using a chord diagram (https://www.bioinformatics.com.cn). Representative subsets of non-redundant gene ontology (GO) terms were summarized and visualized using a clustering algorithm provided by REVIGO (Reduce and Visualize Gene Ontology) version 1.8.1 (http://revigo.irb.hr). Experimental validations of pertinent functions or biological pathways were carried out using many assays as follows.

### Immunoblotting

Cellular proteins were extracted with Laemmli’s buffer before quantifying the protein concentration by Bio-Rad protein assay. An equal amount of proteins derived from each sample was separated by 12% SDS-PAGE. Following electro-transferred onto nitrocellulose membranes, the blots were blocked with 5% non-fat milk/phosphate-buffered saline (PBS) at 25 °C for 1 h and incubated with each of the primary antibodies, which were specific to Snail1 (1:500), GAPDH (1:2000), HSP60 (1:2000), HSP70 (1:2000), DDX1 (1:1000), β-actin (1:2000), p21 (1:250), γH2AX (1:2000), and paxillin (1:1000) and diluted in 1% non-fat milk/PBS, at 4 °C overnight. All primary antibodies were mouse monoclonal and purchased from Santa Cruz Biotechnology, except a rabbit monoclonal anti-Snail1 antibody that was obtained from Cell Signaling Technology. After three washes with PBS, the membranes were incubated with a corresponding secondary antibody conjugated with horseradish peroxidase (Sigma–Aldrich) (1:20,000) at 25 °C for 1 h. Following the final wash, an enhanced chemiluminescence substrate (Thermo Fisher Scientific) was applied to the membranes before autoradiography. Band intensities were measured by ImageQuant TL software (GE Healthcare).

### Immunofluorescence assay

The cells were grown on coverslips for 24 h before immunofluorescence staining. After rinsing with PBS, the cells were fixed and permeabilized with 3.7% formaldehyde/PBS and 0.1% Triton-X100/PBS, respectively (15 min at 25 °C each step). The cells were then blocked in 1% bovine serum albumin (BSA)/PBS at 25 °C for 30 min before incubating at 4 °C overnight with rabbit polyclonal anti-Snail1 (Cell Signaling Technology) (1:100 in 1% BSA/PBS) or each of mouse monoclonal antibodies specific to nucleophosmin, γH2AX, and paxillin (all were from Santa Cruz Biotechnology and diluted 1:50 in 1% BSA/PBS). The secondary antibody used was chicken anti-rabbit IgG conjugated with Alexa Fluor 546 (Invitrogen), donkey-anti-mouse IgG conjugated with Alexa Fluor 555 (Invitrogen), or goat-anti-mouse IgG conjugated with Alexa Fluor 488 (Invitrogen) (all were diluted 1:500 in 1% BSA/PBS at 25 °C for 1 h). Nuclei were counterstained by Hoechst (1:500 in 1% BSA/PBS). Coverslips were mounted onto glass slides using 50% glycerol/PBS. Imaging was done under an Eclipse 80i fluorescence microscope (Nikon). Data acquisition, fluorescence intensity analysis (from at least 100 cells across 10 random fields per sample), and intensity profiling were performed using NIS-Elements BR (version 5.30.03) (Nikon).

### Analysis of nucleolar organizer regions (NORs) by AgNOR staining

The cells were allowed to grow on coverslips for 24 h. AgNOR staining was performed according to a previous study^24^ with modest modification. The cells were rinsed with PBS, fixed with 95% ethanol, and incubated in Carnoy’s solution (absolute ethanol to glacial acetic acid = 3:1) for 30 min. The cells were then rehydrated through (descending) graded alcohols (70%, 50%, and 0%) and stained with AgNO_3_ solution (33% w/v in solution A containing 0.66% w/v gelatin and 0.55% v/v formic acid) at 37 °C in the dark for 30 min. After an intense wash with deionized water, the cells were incubated with 5% w/v sodium thiosulfate for 5 min. Finally, the cells were washed three times with deionized water before mounting onto a glass slide. The NORs were imaged under an Eclipse Ti–S inverted microscope (Nikon). The AgNORs were analyzed by the morphometric method using the ImageJ 1.54g software package (https://imagej.net/ij). Briefly, the images were imported into the software environment, and thresholding was used to create binary images. The AgNORs were then measured by automatically detecting objects found in the binary image using the “Analyze Particles” tool. The total area of AgNORs and their number were quantified relative to the number of nuclei from at least 15 random fields per sample.

### Measurement of cell area after imaging

The cells were seeded into a 6-well plate (4 × 10^5^ cells/well) and incubated for 24 h before imaging under a phase contrast microscope (Nikon). Cell areas were then measured from at least 100 cells in 10 random fields per sample using NIS-Elements D (version 4.11.00) (Nikon).

### Measurement of cell size and cell granularity by flow cytometry

The cells were seeded into a 6-well plate (4 × 10^5^ cells/well) and incubated for 24 h before harvest. After trypsinization, the cells were collected by centrifugation at 300*g* for 3 min, resuspended in 300 μL PBS, and analyzed using the BD Accuri C6 flow cytometer (BD Bioscience). Forward scatter (FSC) and side scatter (SSC) were acquired and averaged from 10,000 cells per sample to represent cell size and granularity, respectively.

### Measurement of matrix metalloproteinase (MMP) production by gelatin zymography

The cells were seeded into a 6-well plate (4 × 10^5^ cells/well) and incubated for 24 h. The cells were then cultured in a serum-free medium for another 24 h. The supernatant was collected and clarified by centrifugation at 300*g* at 4 °C for 5 min. The samples were prepared in a non-reducing buffer without heat before separating in a 10% polyacrylamide gel co-polymerized with gelatin (15 mg). The protein renaturation step was done by washing the gel three times in buffer containing 2.5% Triton X-100, followed by incubating at 37 °C in an incubation buffer (50 mM Tris–HCl (pH 8.0), 10 mM CaCl_2_, and 150 mM NaCl) for 20 h. The enzymatic activities were visualized by Coomassie Blue G-250 staining and appeared as transparent bands over a blue background.

### Statistical analysis

Data were quantified from three independent experiments and were expressed as mean ± standard error of the mean. Statistical tests were performed using PASW Statistics 18 (SPSS Inc.). Mean differences between two independent groups were analyzed using the Student’s *t*-test (when the data were normally distributed) or the Mann–Whitney *U*-test (when the data were not normally distributed). Significant differences were considered when *P*-values < 0.05.

## Results

### Validation of the stably *SNAI1-*overexpressed renal tubular cells

**Figure 1:**
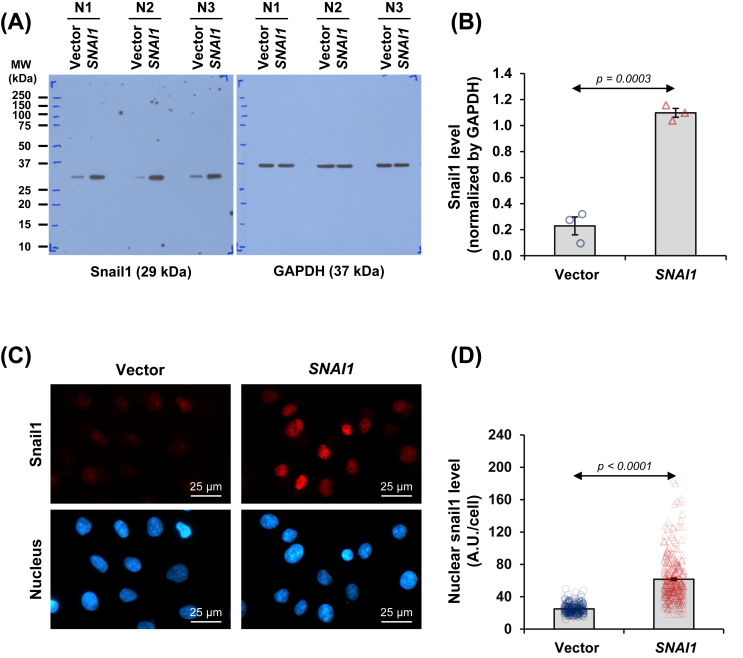
Expression and localization of Snail1 protein in *SNAI1*-overexpressed renal tubular cells. **(A)** Snail1 protein expression was evaluated in vector-control and *SNAI1*-overexpressed HK-2 cells by immunoblotting. **(B)** The Snail1 band intensity was quantified and normalized by that of GAPDH (loading control). **(C)** Expression and localization of Snail1 protein were also examined by immunofluorescence assay. **(D)** The Snail1 fluorescence intensity was evaluated in at least 100 cells across 10 random fields per sample. Each bar shows the mean ± standard error of the mean derived from three independent experiments.

After the generation of stable cells overexpressing *SNAI1* using a retroviral system and selection, the *SNAI1*-overexpressing HK-2 cells were validated for their increased level of Snail1 protein. Immunoblot analysis revealed a substantial increase in Snail1 level in HK-2 cells transduced with the *SNAI1*-containing vector when compared with those transduced with an empty vector (vector-control cells) (Fig. 1A and B). Additionally, the immunofluorescence assay illustrated that the increased Snail1 was mainly in the nucleus (Fig. 1C and D).

### *SNAI1* overexpression induced alterations in the cellular proteome of renal tubular cells

**Figure 2:**
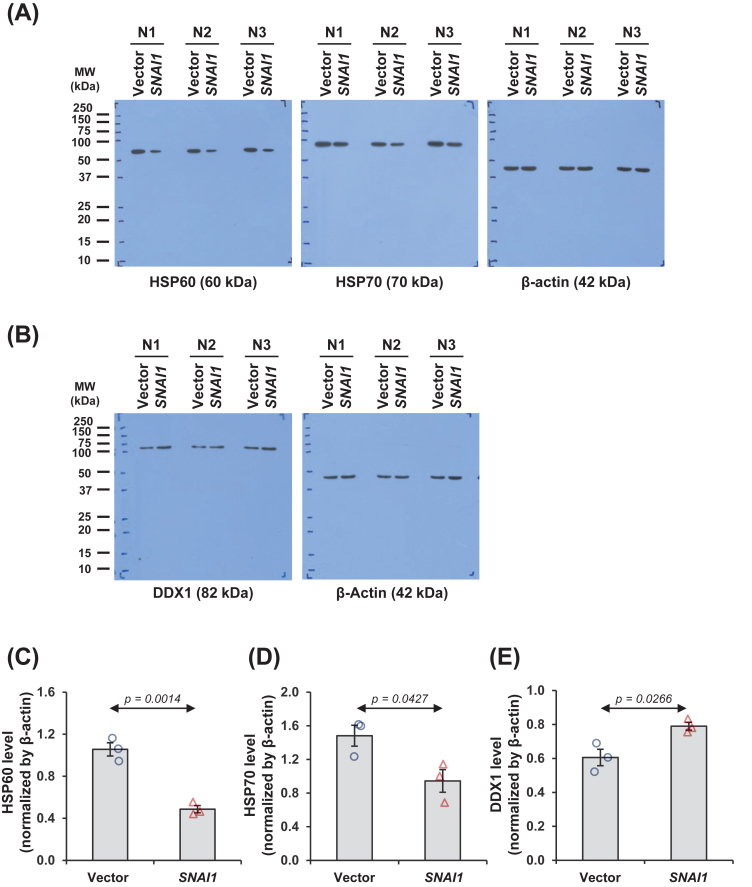
Validation of differentially expressed proteins in *SNAI1*-overexpressed renal tubular cells. **(A, B)** The significantly decreased and increased proteins in *SNAI1*-overexpressed HK-2 cells were validated by immunoblotting compared with vector-control HK-2 cells. **(C**–**E)** Their band intensities were quantified and normalized by that of β-actin (loading control). Each bar shows the mean ± standard error of the mean derived from three independent experiments.

Comparative proteome analysis by the LFQ approach yielded 233 proteins with significantly differential levels between *SNAI1*-overexpressed and vector-control cells (Table 1). To validate the proteome data, some differentially expressed proteins, *i.e.*, heat shock protein 60 (HSP60), heat shock protein 70 (HSP70), and DEAD-box (a motif containing Asp-Glu-Ala-Asp amino acids) helicase 1 (DDX1), were verified by immunoblot analyses. In concordance with LFQ proteomics data, the decreased HSP60 (Fig. 2A and C), decreased HSP70 (Fig. 2A and D), and increased DDX1 (Fig. 2B and E) were observed in *SNAI1*-overexpressed cells compared with vector-control cells.

**Table 1:** Differentially expressed proteins in vector-control versus *SNAI1*-overexpressed HK-2 cells.

Protein name	SwissProt ID	Gene symbol	MS/MS identification score	%Cov	No. of distinct/total matched peptides	MW (kDa)	LFQ intensity (log2)		Δ LFQ intensity (log2)	Adjusted *P*-value
							Vector	*SNAI1*		
10 kDa heat shock protein, mitochondrial	P61604	HSPE1	77.56	47.1	5/5	10.93	24.0886 ± 0.0410	23.3999 ± 0.1008	−0.6887	<0.0001
14 kDa phosphohistidine phosphatase	Q9NRX4	PHPT1	27.61	20.0	2/2	13.83	21.3561 ± 0.0822	21.8051 ± 0.0686	0.4490	0.0004
14-3-3 protein beta/alpha	P31946	YWHAB	187.25	19.9	3/7	28.08	21.4300 ± 0.0924	20.6929 ± 0.1138	−0.7372	0.0002
14-3-3 protein gamma	P61981	YWHAG	188.32	27.5	5/9	28.30	22.6813 ± 0.0522	21.9959 ± 0.1311	−0.6855	0.0001
14-3-3 protein sigma	P31947	SFN	51.94	14.9	2/4	27.77	24.3783 ± 0.0465	24.8528 ± 0.1371	0.4744	0.0029
26S proteasome regulatory subunit 6A	P17980	PSMC3	6.61	2.5	1/1	49.20	19.2063 ± 0.0601	19.9521 ± 0.2128	0.7457	0.0025
26S proteasome regulatory subunit 7	P35998	PSMC2	54.54	5.1	2/2	48.63	20.3344 ± 0.0789	21.1955 ± 0.1105	0.8611	<0.0001
60 kDa heat shock protein, mitochondrial	P10809	HSPD1	323.31	43.1	24/24	61.05	25.0591 ± 0.0220	24.9768 ± 0.0269	−0.0823	0.0205
6-phosphogluconolactonase	O95336	PGLS	12.17	9.7	2/2	27.55	20.4838 ± 0.0415	21.5138 ± 0.1445	1.0300	<0.0001
Acetyl-CoA acetyltransferase, cytosolic	Q9BWD1	ACAT2	32.31	9.3	2/2	41.35	22.1496 ± 0.0581	21.2368 ± 0.0632	−0.9128	<0.0001
Actin, cytoplasmic 2	P63261	ACTG1	323.31	65.9	20/26	41.79	28.5894 ± 0.0272	28.4936 ± 0.0396	−0.0958	0.0466
Actin-related protein 3	P61158	ACTR3	16.10	9.3	2/2	47.37	19.7940 ± 0.0667	20.9089 ± 0.1162	1.1149	<0.0001
Adenylyl cyclase-associated protein 1	Q01518	CAP1	53.87	13.9	5/5	51.90	23.4819 ± 0.0443	23.2126 ± 0.0952	−0.2693	0.0129
ADP/ATP translocase 2	P05141	SLC25A5	22.59	9.4	4/4	32.85	23.4953 ± 0.0540	24.0742 ± 0.0892	0.5789	0.0001
Aldo-keto reductase family 1 member B1	P15121	AKR1B1	81.33	29.1	7/7	35.85	23.0873 ± 0.0778	23.5077 ± 0.1446	0.4204	0.0130
Annexin A5	P08758	ANXA5	163.36	34.4	8/8	35.94	22.2711 ± 0.0753	22.7550 ± 0.2083	0.4839	0.0305
Annexin A7	P20073	ANXA7	36.52	5.3	3/3	52.74	21.0202 ± 0.1313	21.7737 ± 0.1422	0.7535	0.0009
Aspartyl/asparaginyl beta-hydroxylase	Q12797	ASPH	90.30	3.3	1/1	85.86	18.3487 ± 0.1421	19.4734 ± 0.1375	1.1248	0.0001
ATP synthase subunit alpha, mitochondrial	P25705	ATP5F1A	63.21	17.5	8/8	59.75	22.4222 ± 0.0524	21.8276 ± 0.2442	−0.5947	0.0200
ATP synthase subunit beta, mitochondrial	P06576	ATP5F1B	320.46	41.8	13/13	56.56	22.7719 ± 0.0418	22.0677 ± 0.2357	−0.7042	0.0050
ATP-dependent RNA helicase A	Q08211	DHX9	24.13	2.6	3/3	140.96	21.9492 ± 0.0520	21.6175 ± 0.0739	−0.3316	0.0014
ATP-dependent RNA helicase DDX1	Q92499	DDX1	22.78	1.8	1/1	82.43	12.1704 ± 0.2135	17.7609 ± 0.8970	5.5905	0.0001
BAG family molecular chaperone regulator 3	O95817	BAG3	12.04	4.0	2/2	61.59	20.6609 ± 0.1046	20.2146 ± 0.0802	−0.4463	0.0024
B-cell receptor-associated protein 31	P51572	BCAP31	20.57	11.0	3/3	27.99	21.7430 ± 0.1434	21.0909 ± 0.0658	−0.6521	0.0004
Calnexin	P27824	CANX	11.49	4.7	2/2	67.57	22.2472 ± 0.0680	23.0246 ± 0.1296	0.7774	0.0001
Calponin-2	Q99439	CNN2	88.53	17.8	5/5	33.70	20.6254 ± 0.0610	21.6500 ± 0.1651	1.0246	0.0001
Calponin-3	Q15417	CNN3	35.59	10.9	3/3	36.41	22.1910 ± 0.0728	21.4488 ± 0.1362	−0.7423	0.0002
Catenin alpha-1	P35221	CTNNA1	157.35	4.6	3/3	100.07	21.6459 ± 0.0789	21.3307 ± 0.1178	−0.3152	0.0280
Cathepsin D	P07339	CTSD	103.47	16.7	4/4	44.55	21.8704 ± 0.0320	22.3379 ± 0.1064	0.4674	0.0004
CCHC-type zinc finger nucleic acid binding protein	P62633	CNBP	47.72	11.9	2/2	19.46	22.4009 ± 0.0584	22.6560 ± 0.1079	0.2551	0.0385
CCN family member 1	O00622	CCN1	49.08	21.0	7/7	42.03	22.0565 ± 0.0603	23.0953 ± 0.1449	1.0388	<0.0001
CD44 antigen	P16070	CD44	12.69	2.2	2/2	81.54	20.8138 ± 0.0964	21.2394 ± 0.1248	0.4257	0.0090
CD99 antigen	P14209	CD99	7.45	5.4	1/1	18.85	20.4873 ± 0.0399	21.4849 ± 0.1512	0.9975	<0.0001
Cell division control protein 42 homolog	P60953	CDC42	25.00	11.0	1/2	21.26	23.3532 ± 0.0600	23.8782 ± 0.1004	0.5250	0.0004
Charged multivesicular body protein 4b	Q9H444	CHMP4B	33.24	15.6	3/3	24.95	21.7594 ± 0.0628	22.1793 ± 0.1248	0.4199	0.0047
Chloride intracellular channel protein 1	O00299	CLIC1	117.26	31.1	4/4	26.92	22.5276 ± 0.0699	21.7673 ± 0.1975	−0.7603	0.0016
Citrate synthase, mitochondrial	O75390	CS	17.94	6.0	3/3	51.71	22.3480 ± 0.0569	23.0895 ± 0.1263	0.7415	0.0001
Cofilin-1	P23528	CFL1	102.54	31.9	4/6	18.50	24.7159 ± 0.0545	24.3744 ± 0.1322	−0.3414	0.0197
Core histone macro-H2A.1	O75367	MACROH2A1	263.14	8.9	2/2	39.18	21.1328 ± 0.0497	20.4365 ± 0.1978	−0.6963	0.0023
CTP synthase 1	P17812	CTPS1	13.51	4.1	2/2	66.69	21.2069 ± 0.0737	22.5381 ± 0.0828	1.3312	<0.0001
Cysteine-rich protein 2	P52943	CRIP2	53.02	7.7	1/1	22.49	21.3655 ± 0.0977	21.9833 ± 0.0584	0.6179	0.0001
Cytoskeleton-associated protein 4	Q07065	CKAP4	93.32	11.6	6/6	66.02	20.1761 ± 0.1129	21.1302 ± 0.2787	0.9541	0.0034
DAZ-associated protein 1	Q96EP5	DAZAP1	7.08	3.7	1/1	43.38	20.4147 ± 0.0451	20.5941 ± 0.0477	0.1794	0.0084
Deoxyuridine 5-triphosphate nucleotidohydrolase, mitochondrial	P33316	DUT	13.38	7.1	2/2	26.56	22.9973 ± 0.0598	22.7925 ± 0.0774	−0.2048	0.0375
Destrin	P60981	DSTN	15.32	18.8	2/4	18.51	21.3600 ± 0.0835	20.8925 ± 0.1269	−0.4675	0.0041
DNA replication licensing factor MCM3	P25205	MCM3	15.52	1.6	2/2	90.98	20.5356 ± 0.0430	20.8371 ± 0.0873	0.3015	0.0039
DNA replication licensing factor MCM4	P33991	MCM4	12.10	2.5	2/2	96.56	20.7972 ± 0.0650	21.5829 ± 0.1346	0.7857	0.0002
DNA-dependent protein kinase catalytic subunit	P78527	PRKDC	37.35	1.5	5/5	469.08	21.3945 ± 0.1192	20.9386 ± 0.1976	−0.4559	0.0483
Elongation factor 1-beta	P24534	EEF1B2	22.00	9.3	3/3	24.76	22.4575 ± 0.1343	22.0860 ± 0.0893	−0.3716	0.0233
Elongation factor 1-delta	P29692	EEF1D	262.23	17.4	3/3	31.12	23.8922 ± 0.0544	23.4564 ± 0.1439	−0.4359	0.0067
Elongation factor 2	P13639	EEF2	246.27	20.9	17/17	95.34	24.4597 ± 0.0367	24.0604 ± 0.0759	−0.3993	0.0002
Emerin	P50402	EMD	11.85	4.7	1/1	28.99	21.0573 ± 0.0560	22.0094 ± 0.1317	0.9521	<0.0001
Endoplasmic reticulum chaperone BiP	P11021	HSPA5	323.31	24.5	13/14	72.33	23.5054 ± 0.0360	22.3915 ± 0.2471	−1.1139	0.0004
Endoplasmic reticulum resident protein 29	P30040	ERP29	83.33	16.1	3/3	28.99	21.8505 ± 0.0458	21.3204 ± 0.1099	−0.5301	0.0003
Endoplasmin	P14625	HSP90B1	323.31	21.5	14/15	92.47	23.7531 ± 0.0825	24.1162 ± 0.0797	0.3631	0.0035
Enhancer of rudimentary homolog	P84090	ERH	223.12	16.3	1/1	12.26	21.5743 ± 0.0222	20.8074 ± 0.2088	−0.7669	0.0014
Eukaryotic initiation factor 4A-I	P60842	EIF4A1	43.90	10.8	4/4	46.15	22.2235 ± 0.0623	20.4723 ± 0.1669	−1.7512	<0.0001
Eukaryotic translation initiation factor 2 subunit 3	P41091	EIF2S3	54.36	6.8	2/2	51.11	20.8728 ± 0.0604	20.0385 ± 0.1290	−0.8342	0.0001
Eukaryotic translation initiation factor 3 subunit H	O15372	EIF3H	49.74	6.2	3/3	39.93	21.4991 ± 0.0787	21.8318 ± 0.1305	0.3327	0.0306
Eukaryotic translation initiation factor 4 gamma 1	Q04637	EIF4G1	31.60	3.9	5/5	175.49	21.3586 ± 0.0478	20.9430 ± 0.1644	−0.4156	0.0177
Eukaryotic translation initiation factor 4B	P23588	EIF4B	323.31	8.5	4/4	69.15	22.4628 ± 0.0748	22.8193 ± 0.1163	0.3565	0.0126
Eukaryotic translation initiation factor 5A-2	Q9GZV4	EIF5A2	20.15	21.6	3/3	16.79	23.9184 ± 0.1098	24.6271 ± 0.0677	0.7087	0.0001
Ezrin	P15311	EZR	99.75	24.6	11/14	69.41	22.7974 ± 0.0925	23.343 ± 0.0898	0.5456	0.0004
F-actin-capping protein subunit beta	P47756	CAPZB	25.66	14.3	3/3	30.63	21.9508 ± 0.0513	20.8688 ± 0.1388	−1.0820	<0.0001
Far upstream element-binding protein 1	Q96AE4	FUBP1	41.75	12.9	5/8	67.56	21.9184 ± 0.0257	21.4107 ± 0.1653	−0.5078	0.0044
Far upstream element-binding protein 2	Q92945	KHSRP	67.57	13.6	6/9	73.11	22.3571 ± 0.0437	22.5470 ± 0.0600	0.1899	0.0130
Fascin	Q16658	FSCN1	129.31	15.0	7/7	54.53	21.9658 ± 0.0697	21.7688 ± 0.0684	−0.1970	0.0446
Fatty acid synthase	P49327	FASN	198.61	8.4	15/15	273.42	22.2468 ± 0.0407	21.2788 ± 0.1184	−0.9679	<0.0001
Filamin-B	O75369	FLNB	35.39	2.7	5/6	278.16	21.1933 ± 0.2048	22.1240 ± 0.1670	0.9307	0.0017
Fructose-bisphosphate aldolase A	P04075	ALDOA	323.31	55.2	16/21	39.42	25.9593 ± 0.0363	25.7589 ± 0.0873	−0.2004	0.0353
Galectin-1	P09382	LGALS1	181.90	34.8	5/5	14.72	25.0291 ± 0.1427	23.2314 ± 0.3167	−1.7976	0.0002
GDNF family receptor alpha-1	P56159	GFRA1	43.90	9.7	3/3	51.46	20.3057 ± 0.0474	20.8191 ± 0.0957	0.5134	0.0002
Glucose-6-phosphate isomerase	P06744	GPI	50.60	9.1	5/5	63.15	21.8902 ± 0.0734	20.6594 ± 0.1329	−1.2308	<0.0001
Glucosidase 2 subunit beta	P14314	PRKCSH	80.50	12.7	5/5	59.43	22.1405 ± 0.0777	21.3027 ± 0.2859	−0.8378	0.0067
Glutathione S-transferase P	P09211	GSTP1	323.31	24.8	3/3	23.36	23.4844 ± 0.0556	23.0934 ± 0.0983	−0.391	0.0020
Glyceraldehyde-3-phosphate dehydrogenase	P04406	GAPDH	323.31	60.0	17/17	36.05	26.9094 ± 0.0267	26.4652 ± 0.0926	−0.4442	0.0002
GMP synthase [glutamine-hydrolyzing]	P49915	GMPS	20.54	6.6	3/3	76.72	20.6663 ± 0.0696	21.1506 ± 0.1242	0.4843	0.0023
Golgi apparatus protein 1	Q92896	GLG1	94.97	2.9	3/3	134.55	20.8714 ± 0.1054	21.4478 ± 0.0584	0.5764	0.0002
Guanine nucleotide-binding protein G(I)/G(S)/G(O) subunit gamma-12	Q9UBI6	GNG12	12.52	34.7	2/2	8.01	21.6658 ± 0.0526	22.3962 ± 0.1384	0.7304	0.0001
Guanine nucleotide-binding protein G(I)/G(S)/G(T) subunit beta-1	P62873	GNB1	13.26	6.5	2/2	37.38	21.5164 ± 0.0525	20.6852 ± 0.2486	−0.8312	0.0029
Heat shock 70 kDa protein 1B	P0DMV9	HSPA1B	105.96	17.5	2/11	70.05	22.5862 ± 0.0651	21.9598 ± 0.1063	−0.6264	0.0002
Heat shock 70 kDa protein 4	P34932	HSPA4	51.79	10.6	6/8	94.33	21.1053 ± 0.1900	21.8459 ± 0.1395	0.7407	0.0036
Heat shock cognate 71 kDa protein	P11142	HSPA8	323.31	37.9	17/22	70.90	24.9479 ± 0.0278	24.0663 ± 0.1315	−0.8816	<0.0001
Heat shock protein 105 kDa	Q92598	HSPH1	33.74	11.5	5/7	96.86	19.0172 ± 0.2819	21.6055 ± 0.3376	2.5883	0.0001
Heat shock protein beta-1	P04792	HSPB1	323.31	49.3	8/8	22.78	25.9071 ± 0.0343	26.4472 ± 0.0669	0.5401	<0.0001
Heat shock protein HSP 90-alpha	P07900	HSP90AA1	165.20	21.6	10/15	84.66	21.5169 ± 0.0998	20.8474 ± 0.0885	−0.6694	0.0002
Heat shock protein HSP 90-beta	P08238	HSP90AB1	323.31	33.4	20/26	83.26	25.5944 ± 0.0333	25.0860 ± 0.1198	−0.5085	0.0005
Hepatoma-derived growth factor	P51858	HDGF	6.65	3.3	1/1	26.79	20.9681 ± 0.0522	21.5997 ± 0.0884	0.6316	0.0001
Heterogeneous nuclear ribonucleoprotein A/B	Q99729	HNRNPAB	53.97	6.3	2/2	36.22	20.6824 ± 0.0876	21.3928 ± 0.2184	0.7104	0.0045
Heterogeneous nuclear ribonucleoprotein A0	Q13151	HNRNPA0	6.49	4.9	1/1	30.84	19.8282 ± 0.0643	20.7748 ± 0.1993	0.9466	0.0004
Heterogeneous nuclear ribonucleoprotein D-like	O14979	HNRNPDL	49.87	6.7	2/2	46.44	18.1323 ± 0.3229	19.9114 ± 0.1585	1.7792	0.0002
Heterogeneous nuclear ribonucleoprotein H	P31943	HNRNPH1	295.91	12.7	2/4	49.23	21.7797 ± 0.0727	21.4470 ± 0.0856	−0.3327	0.0049
Heterogeneous nuclear ribonucleoprotein K	P61978	HNRNPK	195.66	25.3	10/10	50.98	23.5819 ± 0.0465	23.2403 ± 0.0387	−0.3416	0.0001
Heterogeneous nuclear ribonucleoprotein L	P14866	HNRNPL	67.82	13.8	6/6	64.13	22.8678 ± 0.0791	22.3886 ± 0.1501	−0.4792	0.0067
Heterogeneous nuclear ribonucleoprotein M	P52272	HNRNPM	115.36	22.9	16/16	77.52	23.4177 ± 0.0317	24.2806 ± 0.0597	0.8630	<0.0001
Heterogeneous nuclear ribonucleoprotein R	O43390	HNRNPR	128.42	10.0	2/6	70.94	21.8802 ± 0.1701	21.2051 ± 0.2988	−0.6751	0.0497
Heterogeneous nuclear ribonucleoproteins A2/B1	P22626	HNRNPA2B1	322.16	33.4	10/10	37.43	24.7669 ± 0.0319	23.9734 ± 0.1687	−0.7935	0.0003
Heterogeneous nuclear ribonucleoproteins C1/C2	P07910	HNRNPC	92.26	15.7	4/4	33.67	21.6997 ± 0.2331	24.1765 ± 0.3796	2.4768	0.0001
Histone H1.4	P10412	H1-4	131.82	37.4	7/10	21.87	25.0300 ± 0.0654	23.5608 ± 0.1724	−1.4693	<0.0001
Histone H1.5	P16401	H1-5	115.56	31.9	3/6	22.58	23.0368 ± 0.0752	21.8104 ± 0.2560	−1.2264	0.0002
Histone H2A type 1-J	Q99878	H2AC14	29.70	38.3	1/3	13.94	25.4344 ± 0.0356	24.6680 ± 0.2611	−0.7664	0.0057
Histone H2B type 1-D	P58876	H2BC5	323.31	76.2	1/13	13.94	27.1897 ± 0.0276	27.4154 ± 0.0757	0.2257	0.0071
Histone H2B type 1-H	Q93079	H2BC9	6.89	76.2	1/13	13.89	23.7430 ± 0.0302	24.3647 ± 0.1070	0.6217	0.0001
Histone H2B type 1-N	Q99877	H2BC15	7.30	76.2	1/13	13.92	23.8009 ± 0.0367	24.2698 ± 0.1085	0.4689	0.0005
Histone H4	P62805	H4C1	213.59	62.1	9/9	11.37	26.9571 ± 0.0269	25.8914 ± 0.2075	−1.0657	0.0002
Importin subunit beta-1	Q14974	KPNB1	190.22	5.7	4/4	97.17	22.8426 ± 0.0627	23.2019 ± 0.1246	0.3593	0.0127
Insulin-like growth factor 2 mRNA-binding protein 2	Q9Y6M1	IGF2BP2	11.70	4.2	1/2	66.12	20.9845 ± 0.1437	21.3514 ± 0.0808	0.3669	0.0281
Isoleucine--tRNA ligase, mitochondrial	Q9NSE4	IARS2	9.35	1.2	1/1	113.79	20.8940 ± 0.0730	20.6426 ± 0.0667	−0.2514	0.0134
Lamina-associated polypeptide 2, isoforms beta/gamma	P42167	TMPO	50.30	13.2	5/5	50.67	20.0793 ± 0.2333	22.2947 ± 0.1740	2.2154	<0.0001
Lamin-B1	P20700	LMNB1	53.35	8.9	4/4	66.41	21.9562 ± 0.0385	22.6386 ± 0.1626	0.6824	0.0005
l-aminoadipate-semialdehyde dehydrogenase-phosphopantetheinyl transferase	Q9NRN7	AASDHPPT	8.08	4.9	1/1	35.78	20.8561 ± 0.1051	19.2206 ± 0.0763	−1.6354	<0.0001
Large ribosomal subunit protein eL13	P26373	RPL13	142.64	25.1	7/7	24.26	22.9119 ± 0.0406	22.4705 ± 0.1066	−0.4414	0.0009
Large ribosomal subunit protein eL22	P35268	RPL22	86.47	31.2	3/3	14.79	23.2677 ± 0.0768	22.7261 ± 0.2428	−0.5416	0.0346
Large ribosomal subunit protein eL24	P83731	RPL24	30.86	26.1	5/5	17.78	21.5987 ± 0.0621	22.0954 ± 0.0992	0.4967	0.0004
Large ribosomal subunit protein eL34	P49207	RPL34	26.63	31.6	4/4	13.29	23.2107 ± 0.0292	23.7266 ± 0.1325	0.5159	0.0011
Large ribosomal subunit protein eL42	P83881	RPL36A	9.71	9.4	1/1	12.44	21.4075 ± 0.0758	21.1345 ± 0.0728	−0.2730	0.0119
Large ribosomal subunit protein eL6	Q02878	RPL6	30.88	13.9	5/5	32.73	23.5619 ± 0.0771	24.0234 ± 0.0895	0.4615	0.0008
Large ribosomal subunit protein eL8	P62424	RPL7A	61.64	12.8	3/3	30.00	23.3284 ± 0.0473	22.3746 ± 0.2707	−0.9538	0.0019
Large ribosomal subunit protein uL11	P30050	RPL12	55.14	28.5	3/3	17.82	24.5036 ± 0.0471	24.7305 ± 0.0699	0.2269	0.0091
Large ribosomal subunit protein uL2	P62917	RPL8	85.93	10.5	2/2	28.02	23.9031 ± 0.0296	23.4894 ± 0.1618	−0.4137	0.0143
Large ribosomal subunit protein uL23	P62750	RPL23A	38.55	17.9	3/3	17.70	22.3844 ± 0.0877	22.7353 ± 0.1440	0.3509	0.0386
Large ribosomal subunit protein uL3	P39023	RPL3	22.69	12.9	4/4	46.11	21.6962 ± 0.0664	21.8963 ± 0.0585	0.2001	0.0256
Large ribosomal subunit protein uL30	P18124	RPL7	38.97	16.9	3/3	29.23	22.6523 ± 0.0686	21.9230 ± 0.1384	−0.7293	0.0002
Large ribosomal subunit protein uL4	P36578	RPL4	53.85	9.8	5/5	47.70	23.8087 ± 0.0368	24.1438 ± 0.1059	0.3351	0.0048
Large ribosomal subunit protein uL5	P62913	RPL11	99.18	18.0	4/4	20.25	22.5357 ± 0.0876	21.4039 ± 0.1998	−1.1318	0.0002
Leucine-rich repeat-containing protein 59	Q96AG4	LRRC59	65.99	7.2	2/2	34.93	20.7251 ± 0.0685	21.0354 ± 0.1246	0.3102	0.0306
LIM and SH3 domain protein 1	Q14847	LASP1	87.92	29.5	7/7	29.72	22.2531 ± 0.0617	21.9006 ± 0.1636	−0.3525	0.0445
l-lactate dehydrogenase B chain	P07195	LDHB	177.76	34.1	7/9	36.64	25.5091 ± 0.0249	25.0317 ± 0.1165	−0.4774	0.0005
Macrophage-capping protein	P40121	CAPG	89.55	7.2	2/2	38.50	22.6236 ± 0.0599	23.2662 ± 0.1202	0.6426	0.0002
Metallothionein-2	P02795	MT2A	70.42	49.2	1/3	6.04	21.4732 ± 0.0603	22.6522 ± 0.0853	1.1790	<0.0001
Methylated-DNA--protein-cysteine methyltransferase	P16455	MGMT	23.51	6.8	1/1	21.65	18.7652 ± 0.0899	19.4331 ± 0.0883	0.6680	0.0002
Microtubule-associated protein 4	P27816	MAP4	56.09	6.3	5/5	121.00	22.4217 ± 0.0629	22.6198 ± 0.0309	0.1981	0.0067
Microtubule-associated protein RP/EB family member 1	Q15691	MAPRE1	68.38	17.2	3/3	30.00	23.0041 ± 0.0524	22.3450 ± 0.2624	−0.6591	0.0164
Midkine	P21741	MDK	31.96	14.0	2/2	15.59	20.5629 ± 0.0845	21.0801 ± 0.1001	0.5172	0.0007
Moesin	P26038	MSN	182.39	38.6	15/18	67.82	24.6059 ± 0.0348	24.2905 ± 0.0831	−0.3154	0.0018
Muscleblind-like protein 1	Q9NR56	MBNL1	6.50	2.8	1/1	41.82	17.7769 ± 0.2872	20.3653 ± 0.1499	2.5884	<0.0001
Myosin-9	P35579	MYH9	323.31	15.5	21/27	226.53	23.2671 ± 0.0556	24.1622 ± 0.1044	0.8951	<0.0001
Nascent polypeptide-associated complex subunit alpha	Q13765	NACA	106.81	21.9	3/3	23.38	22.8003 ± 0.0461	21.3297 ± 0.2573	−1.4706	0.0001
Neuroblast differentiation-associated protein AHNAK	Q09666	AHNAK	113.9	5.5	14/14	629.09	21.1444 ± 0.0935	21.8198 ± 0.1987	0.6754	0.0041
Non-histone chromosomal protein HMG-17	P05204	HMGN2	71.76	34.4	2/2	9.39	21.4248 ± 0.1017	21.9737 ± 0.1449	0.5489	0.0039
Non-POU domain-containing octamer-binding protein	Q15233	NONO	57.73	15.5	6/6	54.23	22.1951 ± 0.0748	21.0005 ± 0.1265	−1.1946	<0.0001
Nuclear migration protein nudC	Q9Y266	NUDC	6.94	3.3	1/1	38.24	22.0505 ± 0.0601	22.5273 ± 0.1449	0.4768	0.0045
Nucleolin	P19338	NCL	44.66	7.9	5/5	76.61	21.5457 ± 0.1251	22.5725 ± 0.1142	1.0268	0.0001
Nucleophosmin	P06748	NPM1	134.46	20.7	7/7	32.58	24.5321 ± 0.0829	24.8015 ± 0.0886	0.2694	0.0280
Paxillin	P49023	PXN	80.66	2.9	1/1	64.51	19.7962 ± 0.0638	20.5844 ± 0.1839	0.7881	0.0005
PDZ and LIM domain protein 1	O00151	PDLIM1	85.61	35.0	6/6	36.07	22.0799 ± 0.0693	22.9594 ± 0.0367	0.8795	<0.0001
Peptidyl-prolyl cis–trans isomerase FKBP1A	P62942	FKBP1A	12.52	12.0	1/1	11.95	23.4834 ± 0.0555	23.8459 ± 0.0473	0.3625	0.0002
Peroxiredoxin-6	P30041	PRDX6	104.87	32.6	5/5	25.04	22.6455 ± 0.0876	22.2425 ± 0.1392	−0.4029	0.0168
Phosphoglycerate kinase 1	P00558	PGK1	323.31	48.9	17/17	44.61	24.4540 ± 0.0339	24.3401 ± 0.0172	−0.1139	0.0047
Phosphoglycerate mutase 1	P18669	PGAM1	246.18	51.2	11/11	28.8	24.0265 ± 0.1085	24.7715 ± 0.0296	0.7450	<0.0001
Plastin-3	P13797	PLS3	178.05	15.6	7/7	70.81	21.9644 ± 0.0575	23.1371 ± 0.2133	1.1727	0.0002
Polypyrimidine tract-binding protein 1	P26599	PTBP1	85.81	11.3	4/6	59.63	21.9349 ± 0.0984	20.9772 ± 0.1720	−0.9577	0.0001
Probable ATP-dependent RNA helicase DDX17	Q92841	DDX17	72.35	5.6	3/3	80.27	22.1660 ± 0.0647	22.5890 ± 0.0485	0.4230	0.0002
Procollagen-lysine,2-oxoglutarate 5-dioxygenase 2	O00469	PLOD2	19.02	1.9	1/1	84.69	21.4483 ± 0.0814	21.9815 ± 0.2165	0.5332	0.0234
Profilin-1	P07737	PFN1	48.76	36.4	5/5	15.05	23.1788 ± 0.0411	21.7243 ± 0.4225	−1.4545	0.0022
Profilin-2	P35080	PFN2	17.36	10.0	1/1	15.05	21.6797 ± 0.0892	21.1321 ± 0.0727	−0.5476	0.0002
Programmed cell death protein 5	O14737	PDCD5	38.14	10.4	1/1	14.29	20.7905 ± 0.0628	20.2595 ± 0.0839	−0.5310	0.0002
Prohibitin-2	Q99623	PHB2	29.66	15.1	5/5	33.30	21.2001 ± 0.1695	22.8315 ± 0.1167	1.6314	<0.0001
Prolyl 3-hydroxylase 1	Q32P28	P3H1	15.67	1.6	1/1	83.39	20.1645 ± 0.0867	20.6024 ± 0.0858	0.4378	0.0016
Prostaglandin E synthase 3	Q15185	PTGES3	23.23	10.6	2/2	18.70	22.1711 ± 0.0504	21.1888 ± 0.1862	−0.9823	0.0002
Proteasome subunit alpha type-6	P60900	PSMA6	100.6	16.7	3/3	27.40	21.4438 ± 0.0887	21.1655 ± 0.0883	−0.2783	0.0281
Protein disulfide-isomerase A6	Q15084	PDIA6	87.18	20.0	7/7	48.12	22.0603 ± 0.0491	21.2593 ± 0.1199	−0.8010	0.0001
Protein PTHB1	Q3SYG4	BBS9	7.07	2.6	1/1	99.28	24.4797 ± 0.0980	24.9101 ± 0.0600	0.4304	0.0013
Protein S100-A11	P31949	S100A11	284.91	56.2	5/5	11.74	24.2217 ± 0.0572	23.3496 ± 0.1539	−0.872	0.0001
Protein transport protein Sec23A	Q15436	SEC23A	38.56	4.7	3/3	86.16	19.5551 ± 0.0930	19.9653 ± 0.1001	0.4102	0.0046
Protein transport protein Sec61 subunit beta	P60468	SEC61B	8.74	15.6	1/1	9.97	21.0813 ± 0.0934	22.1951 ± 0.1023	1.1138	<0.0001
Prothymosin alpha	P06454	PTMA	146.00	12.6	1/1	12.20	21.7979 ± 0.0972	21.4125 ± 0.0760	−0.3854	0.0038
Putative elongation factor 1-alpha-like 3	Q5VTE0	EEF1A1P5	142.83	32.3	3/11	50.18	26.4043 ± 0.0479	26.1990 ± 0.0567	−0.2054	0.0078
Putative nucleoside diphosphate kinase	O60361	NME2P1	187.94	26.3	3/3	15.53	24.9093 ± 0.0806	23.5100 ± 0.2607	−1.3992	0.0002
Putative ribosomal protein eS26-like	Q5JNZ5	RPS26P11	11.63	13.9	2/2	13.00	21.0978 ± 0.2176	22.3541 ± 0.4683	1.2563	0.0175
Putative ribosomal protein uL10-like	Q8NHW5	RPLP0P6	60.21	21.5	5/5	34.36	23.2273 ± 0.0478	21.4801 ± 0.2421	−1.7472	<0.0001
Rab GDP dissociation inhibitor beta	P50395	GDI2	103.38	16.6	7/7	50.66	21.3819 ± 0.0645	22.0381 ± 0.1694	0.6562	0.0016
Ras-related C3 botulinum toxin substrate 1	P63000	RAC1	7.43	10.9	1/2	21.45	21.8764 ± 0.0611	21.1996 ± 0.2817	−0.6767	0.0212
Ras-related protein Rab-1A	P62820	RAB1A	12.00	7.8	2/2	22.68	21.1872 ± 0.0945	21.9938 ± 0.1704	0.8067	0.0004
Ras-related protein Rab-5C	P51148	RAB5C	9.12	5.6	1/1	23.48	19.9361 ± 0.0786	20.2874 ± 0.1021	0.3513	0.0084
Ras-related protein R-Ras2	P62070	RRAS2	15.84	5.9	1/1	23.40	20.1248 ± 0.0943	20.5961 ± 0.0912	0.4712	0.0017
Replication protein A 70 kDa DNA-binding subunit	P27694	RPA1	17.41	2.6	1/1	68.14	19.9205 ± 0.1317	19.5491 ± 0.0583	−0.3714	0.0126
Ribosome-binding protein 1	Q9P2E9	RRBP1	58.47	18.8	9/9	152.45	18.9249 ± 0.3691	22.0408 ± 0.1365	3.1158	<0.0001
Septin-7	Q16181	SEPTIN7	21.50	6.4	3/3	50.68	21.7624 ± 0.0654	22.1978 ± 0.1077	0.4353	0.0021
Serine hydroxymethyltransferase, mitochondrial	P34897	SHMT2	20.75	6.7	3/3	55.99	22.6197 ± 0.1713	21.4923 ± 0.3584	−1.1274	0.0067
Serine/threonine-protein phosphatase 2A 65 kDa regulatory subunit A alpha isoform	P30153	PPP2R1A	21.34	70.0	3/3	65.31	22.0992 ± 0.0743	22.4454 ± 0.0792	0.3463	0.0033
Serine/threonine-protein phosphatase PP1-gamma catalytic subunit	P36873	PPP1CC	18.02	3.1	1/1	36.98	22.3454 ± 0.0722	20.7779 ± 0.1062	−1.5675	<0.0001
Serpin H1	P50454	SERPINH1	69.47	23.7	6/6	46.44	21.2231 ± 0.0944	21.5235 ± 0.1177	0.3004	0.0467
SERPINE1 mRNA-binding protein 1	Q8NC51	SERBP1	310.86	15.0	4/4	44.97	24.5105 ± 0.0575	24.2153 ± 0.0507	−0.2952	0.0010
SH3 domain-binding glutamic acid-rich-like protein 3	Q9H299	SH3BGRL3	30.42	43.0	4/4	10.44	21.9567 ± 0.1125	22.7367 ± 0.1722	0.7800	0.0011
Signal peptidase complex subunit 2	Q15005	SPCS2	118.14	9.7	1/1	25.00	19.3569 ± 0.1134	19.8600 ± 0.1536	0.5031	0.0109
Signal transducer and activator of transcription 1-alpha/beta	P42224	STAT1	59.72	11.6	8/8	87.33	22.5811 ± 0.0788	21.1575 ± 0.1115	−1.4236	<0.0001
Small nuclear ribonucleoprotein F	P62306	SNRPF	28.79	15.1	1/1	9.73	21.1205 ± 0.0720	21.4133 ± 0.0708	0.2928	0.0057
Small ribosomal subunit protein eS1	P61247	RPS3A	180.34	33.0	10/10	29.95	23.7545 ± 0.0539	22.9775 ± 0.0803	−0.7770	<0.0001
Small ribosomal subunit protein eS10	P46783	RPS10	11.66	11.5	2/2	18.9	20.9981 ± 0.2817	22.4936 ± 0.5080	1.4955	0.0127
Small ribosomal subunit protein eS17	P08708	RPS17	12.68	25.2	2/2	15.55	20.3059 ± 0.2502	21.0287 ± 0.1876	0.7228	0.0230
Small ribosomal subunit protein eS21	P63220	RPS21	34.03	27.7	4/4	9.11	22.2630 ± 0.0312	22.9402 ± 0.0931	0.6772	<0.0001
Small ribosomal subunit protein eS25	P62851	RPS25	104.37	24.8	3/3	13.74	23.1170 ± 0.0963	23.6053 ± 0.1396	0.4883	0.0059
Small ribosomal subunit protein eS28	P62857	RPS28	50.67	29.0	2/2	7.84	21.5280 ± 0.1821	22.4192 ± 0.1256	0.8912	0.0005
Small ribosomal subunit protein eS6	P62753	RPS6	51.93	20.1	4/4	28.68	23.5973 ± 0.0587	23.1781 ± 0.0639	−0.4192	0.0001
Small ribosomal subunit protein eS8	P62241	RPS8	110.32	24.0	4/4	24.21	21.9108 ± 0.0392	21.2360 ± 0.0981	−0.6749	<0.0001
Small ribosomal subunit protein uS11	P62263	RPS14	20.59	19.2	3/3	16.27	23.7512 ± 0.0672	24.2578 ± 0.0937	0.5066	0.0003
Small ribosomal subunit protein uS15	P62277	RPS13	22.40	18.5	3/3	17.22	22.3994 ± 0.1483	21.8313 ± 0.0559	−0.5680	0.0016
Small ribosomal subunit protein uS17	P62280	RPS11	46.80	19.6	3/3	18.43	23.3263 ± 0.0698	22.5811 ± 0.1631	−0.7452	0.0004
Small ribosomal subunit protein uS2	P08865	RPSA	84.09	25.8	5/5	32.85	23.3450 ± 0.0793	22.6992 ± 0.2228	−0.6459	0.0084
Small ribosomal subunit protein uS3	P23396	RPS3	112.15	30.0	6/6	26.69	23.3517 ± 0.0376	23.1603 ± 0.0570	−0.1913	0.0071
Small ribosomal subunit protein uS5	P15880	RPS2	55.04	23.9	7/7	31.32	23.5862 ± 0.0403	24.1154 ± 0.0781	0.5291	0.0001
Small ribosomal subunit protein uS7	P46782	RPS5	153.86	24.5	5/5	22.88	23.1473 ± 0.0891	22.1469 ± 0.1760	−1.0004	0.0002
Small ubiquitin-related modifier 4	Q6EEV6	SUMO4	60.54	12.6	1/1	10.65	23.8876 ± 0.0617	23.5526 ± 0.0840	−0.3350	0.0032
Solute carrier family 25 member 3	Q00325	SLC25A3	31.36	5.8	2/2	40.09	23.5826 ± 0.0663	23.1758 ± 0.1206	−0.4069	0.0050
Sorting nexin-3	O60493	SNX3	12.16	11.1	2/2	18.76	22.3891 ± 0.0859	20.9681 ± 0.2171	−1.421	0.0001
Splicing factor 3B subunit 5	Q9BWJ5	SF3B5	8.49	17.4	1/1	10.14	21.2720 ± 0.0477	21.6660 ± 0.0741	0.3941	0.0004
Splicing factor, proline- and glutamine-rich	P23246	SFPQ	47.18	5.7	4/4	76.15	23.2209 ± 0.0655	23.0147 ± 0.0582	−0.2062	0.0210
Staphylococcal nuclease domain-containing protein 1	Q7KZF4	SND1	124.70	10.3	7/7	102.00	22.4397 ± 0.0680	23.0240 ± 0.0756	0.5843	0.0001
Stathmin-2	Q93045	STMN2	7.68	15.1	1/3	20.83	23.2180 ± 0.0470	23.8684 ± 0.1284	0.6504	0.0002
Stress-70 protein, mitochondrial	P38646	HSPA9	158.15	16.1	8/8	73.68	23.2279 ± 0.0371	22.8697 ± 0.1619	−0.3582	0.0326
Stress-induced-phosphoprotein 1	P31948	STIP1	12.11	3.7	2/2	62.64	21.3637 ± 0.1565	22.2538 ± 0.3167	0.8901	0.0142
Talin-1	Q9Y490	TLN1	145.44	5.0	9/9	269.76	21.2471 ± 0.0388	21.4372 ± 0.0640	0.1901	0.0134
T-complex protein 1 subunit epsilon	P48643	CCT5	34.8	10.2	4/4	59.67	22.9871 ± 0.0830	22.0760 ± 0.1497	−0.9111	0.0001
T-complex protein 1 subunit zeta	P40227	CCT6A	125.33	26.4	11/11	58.02	22.2165 ± 0.1205	21.3795 ± 0.1318	−0.8370	0.0002
Thioredoxin domain-containing protein 5	Q8NBS9	TXNDC5	138.16	15.3	5/5	47.63	22.8044 ± 0.0377	23.1553 ± 0.0611	0.3509	0.0001
Transaldolase	P37837	TALDO1	82.09	24.9	10/10	37.54	22.6744 ± 0.0962	22.0812 ± 0.1532	−0.5933	0.0029
Transcription elongation factor A protein 1	P23193	TCEA1	62.75	15.0	4/4	33.97	21.6455 ± 0.0740	21.9839 ± 0.0851	0.3384	0.0046
Transferrin receptor protein 1	P02786	TFRC	13.03	3.4	2/2	84.87	21.6505 ± 0.0928	20.7295 ± 0.2367	−0.9209	0.0016
Transforming growth factor-beta-induced protein ig-h3	Q15582	TGFBI	26.85	11.0	4/4	74.68	22.4362 ± 0.0721	21.7080 ± 0.0814	−0.7282	<0.0001
Transgelin-2	P37802	TAGLN2	323.31	70.4	13/13	22.39	24.7196 ± 0.0288	24.2683 ± 0.0606	−0.4513	<0.0001
Translocon-associated protein subunit delta	P51571	SSR4	17.93	24.3	3/3	19.00	21.4548 ± 0.1018	20.4767 ± 0.1310	−0.9782	0.0001
Transmembrane protein 245	Q9H330	TMEM245	9.98	1.5	1/1	97.36	19.7231 ± 0.0427	20.2986 ± 0.1431	0.5755	0.0010
Triosephosphate isomerase	P60174	TPI1	323.31	63.1	14/14	26.67	25.8246 ± 0.0338	25.6240 ± 0.0623	−0.2005	0.0068
Tubulin beta-4B chain	P68371	TUBB4B	323.31	38.4	3/18	49.83	26.5366 ± 0.0316	26.0839 ± 0.0355	−0.4526	<0.0001
Tubulin beta-8 chain	Q3ZCM7	TUBB8	36.79	21.6	1/8	49.78	23.2576 ± 0.1783	22.4405 ± 0.3665	−0.8171	0.0454
Ubiquitin-associated protein 2-like	Q14157	UBAP2L	29.22	2.4	2/2	114.53	21.0050 ± 0.0883	20.7085 ± 0.0478	−0.2965	0.0050
Vesicle-trafficking protein SEC22b	O75396	SEC22B	27.85	24.2	3/3	24.74	19.9190 ± 0.1480	20.9922 ± 0.0627	1.0732	<0.0001
Vimentin	P08670	VIM	323.31	56.0	25/28	53.65	26.1340 ± 0.0244	25.4974 ± 0.1484	−0.6365	0.0004
Voltage-dependent anion-selective channel protein 1	P21796	VDAC1	10.62	4.2	1/1	30.77	22.6045 ± 0.0394	21.9128 ± 0.2411	−0.6916	0.0067
Y-box-binding protein 1	P67809	YBX1	105.58	26.2	3/3	35.92	20.8297 ± 0.0825	21.5083 ± 0.1156	0.6786	0.0002
YTH domain-containing family protein 1	Q9BYJ9	YTHDF1	7.70	2.3	1/1	60.87	19.7169 ± 0.0460	19.4649 ± 0.1081	−0.2521	0.0333
Zyxin	Q15942	ZYX	103.38	23.4	8/8	61.28	21.0872 ± 0.0725	21.7611 ± 0.0933	0.6739	0.0001

Note: %Cov represents the percentage of the ratio of the number of identified amino acid residues to the total number of amino acid residues in the protein sequence. Δ LFQ intensity (log2) represents the degree of differential expression (a positive value indicates an increase, whereas a negative value indicates a decrease in protein expression in the *SNAI1*-overexpressed cells). MW, molecular weight; LFQ, label-free quantification.

### Deciphering the biological significance of the altered cellular proteome

**Figure 3:**
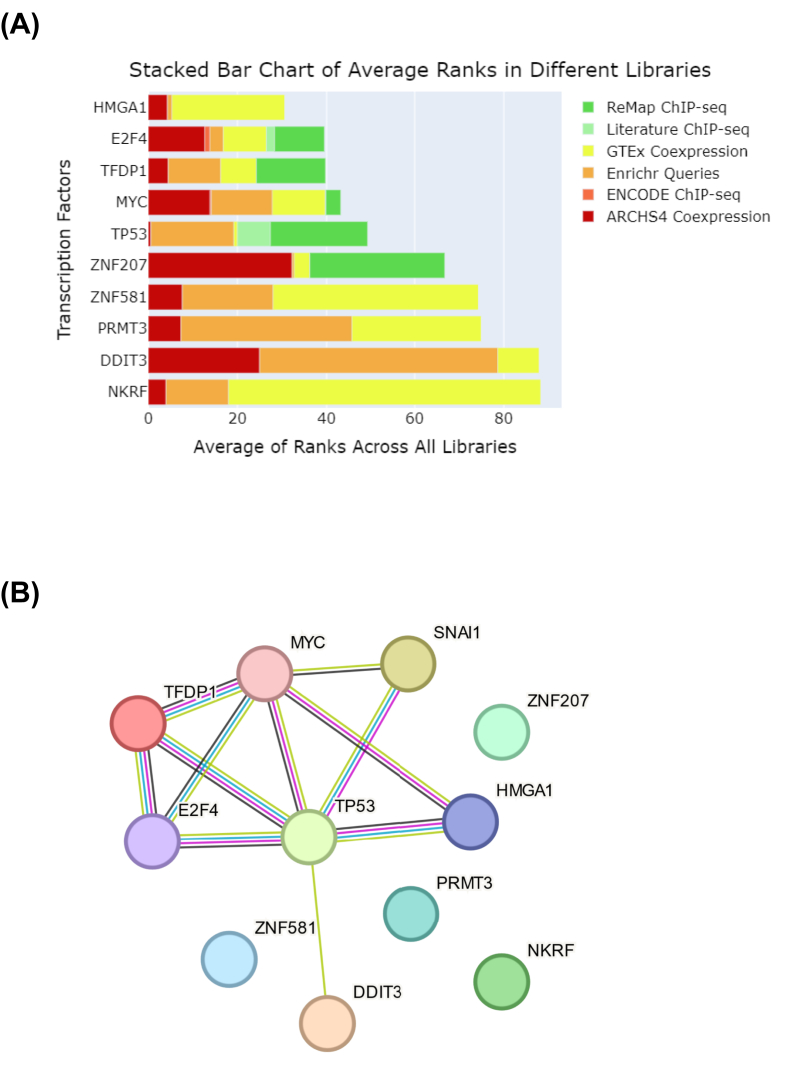
Top 10 transcription factors relevant to *SNAI1*-induced changes in the cellular proteome of renal tubular cells. **(A)** The X2K Appyter (Expression2Kinases) was utilized to predict the top 10 transcription factors relevant to *SNAI1* overexpression. The stacked bar plot represents the average rank of each transcription factor across different libraries (displayed as segments in different colors). **(B)** The network depicting interactions among these top 10 transcription factors together with Snail1 was visualized using the STRING database.

X2K Appyter (Expression2Kinases) was utilized to gain insights into the upstream regulatory transcription factors responsible for the alterations in the cellular proteome induced by ectopic *SNAI1* expression. The top 10 transcription factors induced by *SNAI1* overexpression were ranked based on various libraries and algorithms and were displayed by a stacked bar plot of the average rank across libraries (Fig. 3A). These transcription factors were HMGA1, E2F4, TFDP1, MYC, TP53, ZNF207, ZNF581, PRMT3, DDIT3, and NKRF. Downstream proteins regulated by these top 10 transcription factors are summarized in Table S1. As Snail1 is essential for regulating multiple genes involved in cell reprogramming and EMT, we examined how Snail1 interacted with these transcription factors. Their interacting network obtained via the STRING database revealed that Snail1 acted in concert with six of these top 10 transcription factors, but had direct interactions with only MYC and TP53 (Fig. 3B).

**Figure 4:**
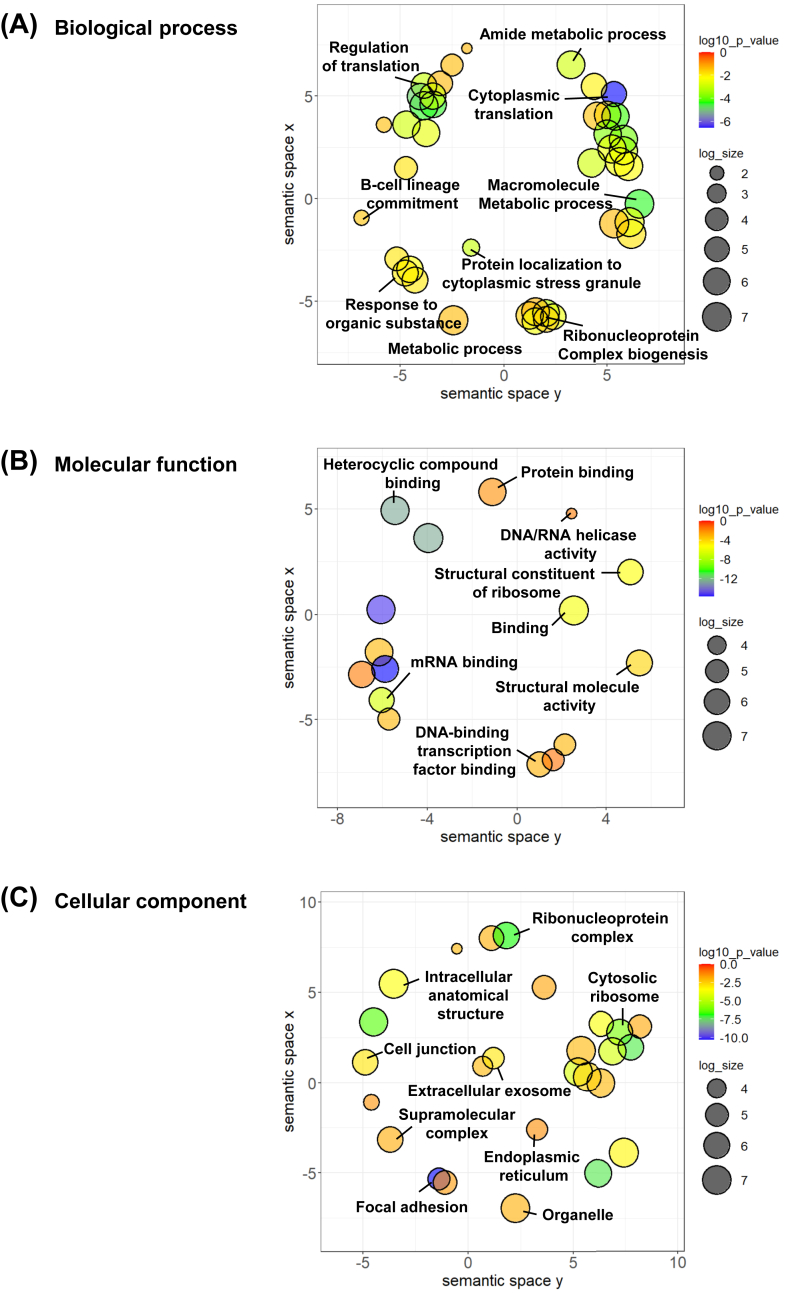
Enrichment analyses of significantly altered proteins induced by *SNAI1* overexpression. Enrichment analyses were done for **(A)** biological process, **(B)** molecular function, and **(C)** cellular component based on the STRING database. The REVIGO tool was utilized to summarize and visualize representative subsets of non-redundant gene ontology (GO) terms. The *x* and *y* axes represent the coordinates of GO terms in a 2D space created by multivariate dimensionality reduction techniques. These coordinates were derived from the semantic similarity between GO terms. Adjusted *P*-values from the hypergeometric test were subjected to a log10 transformation. Differences in color shade and size of individual nodes indicate the statistically significant level and frequency, respectively, of the GO terms.

**Figure 5:**
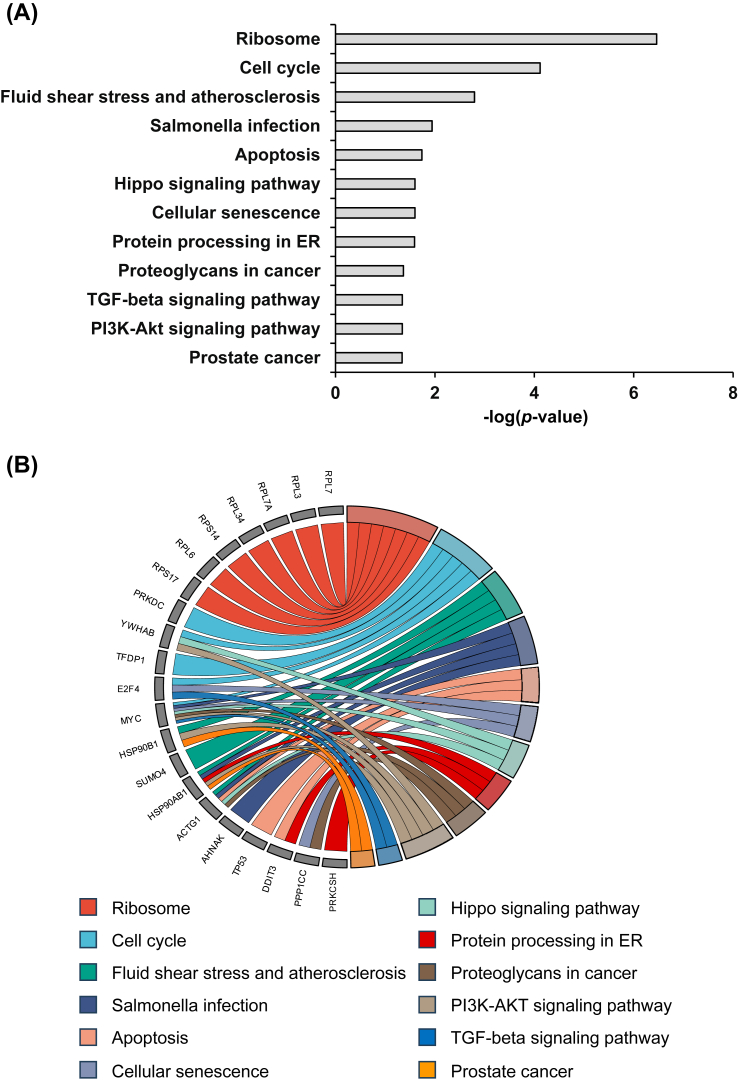
Deciphering the biological significance of the altered cellular proteome by KEGG pathway analysis. KEGG pathway analysis was done to highlight the biological significance of the altered cellular proteome. **(A)** The key pathways induced by *SNAI1* overexpression. Adjusted *P*-values from the hypergeometric test were subjected to a –log10 transformation. **(B)** The connection between differentially expressed proteins and various KEGG pathways was depicted using a chord diagram.

Additionally, the downstream proteins regulated by the predicted transcription factors were further subjected to GO or functional enrichment analyses. The key biological processes enriched included regulation of translation, metabolic process, and ribonucleoprotein complex biogenesis (Fig. 4A). The key molecular functions enriched were mRNA binding, heterocyclic compound binding, structural constituent of ribosome, DNA binding, and transcription factor binding (Fig. 4B). The prominent cellular components involved included focal adhesion, ribonucleoprotein complex, and cytosolic ribosome (Fig. 4C). Moreover, KEGG pathway analysis revealed several pathways that were induced by ectopic *SNAI1* expression, including ribosome, cell cycle, apoptosis, Hippo signaling, cellular senescence, protein processing in endoplasmic reticulum, TGF-β signaling pathway, and PI3K-Akt signaling pathways (Fig. 5A). The relationship and overlaps between proteins and the enriched pathways are illustrated in Figure 5B.

### *SNAI1* overexpression induced ribosome biogenesis in renal tubular cells

**Figure 6:**
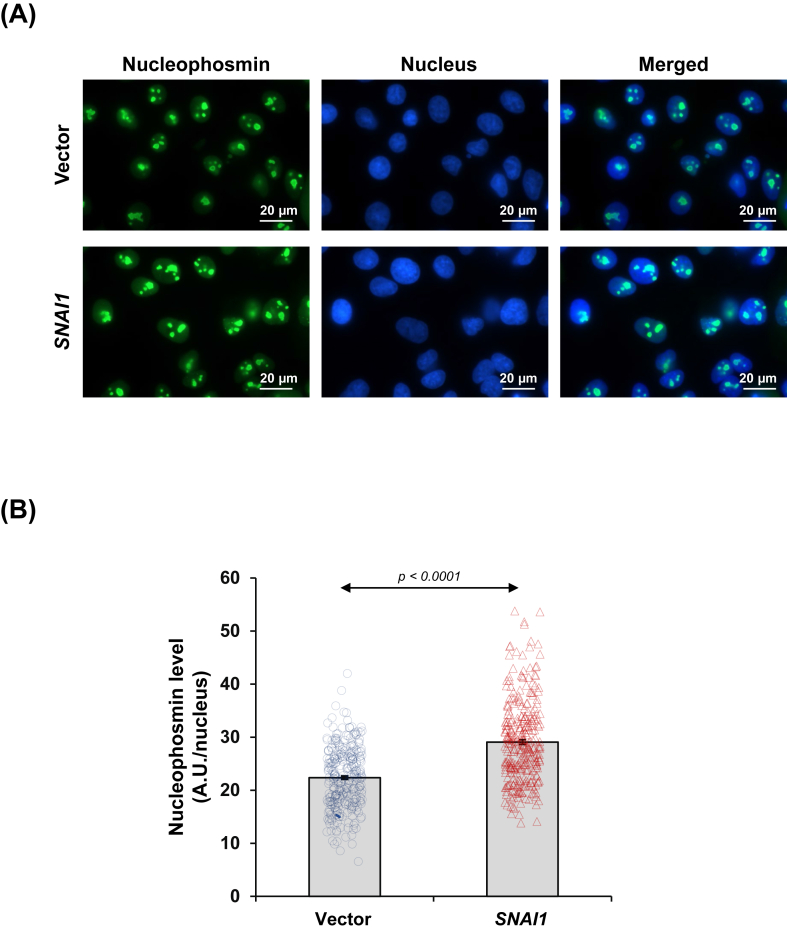
*SNAI1* overexpression up-regulated nucleophosmin expression in renal tubular cells. **(A)** Expression and localization of nucleophosmin in vector-control and *SNAI1*-overexpressed HK-2 cells were examined by immunofluorescence assay. **(B)** The nucleophosmin fluorescence intensity was evaluated in at least 100 cells across 10 random fields per sample. Each bar shows the mean ± standard error of the mean derived from three independent experiments.

**Figure 7:**
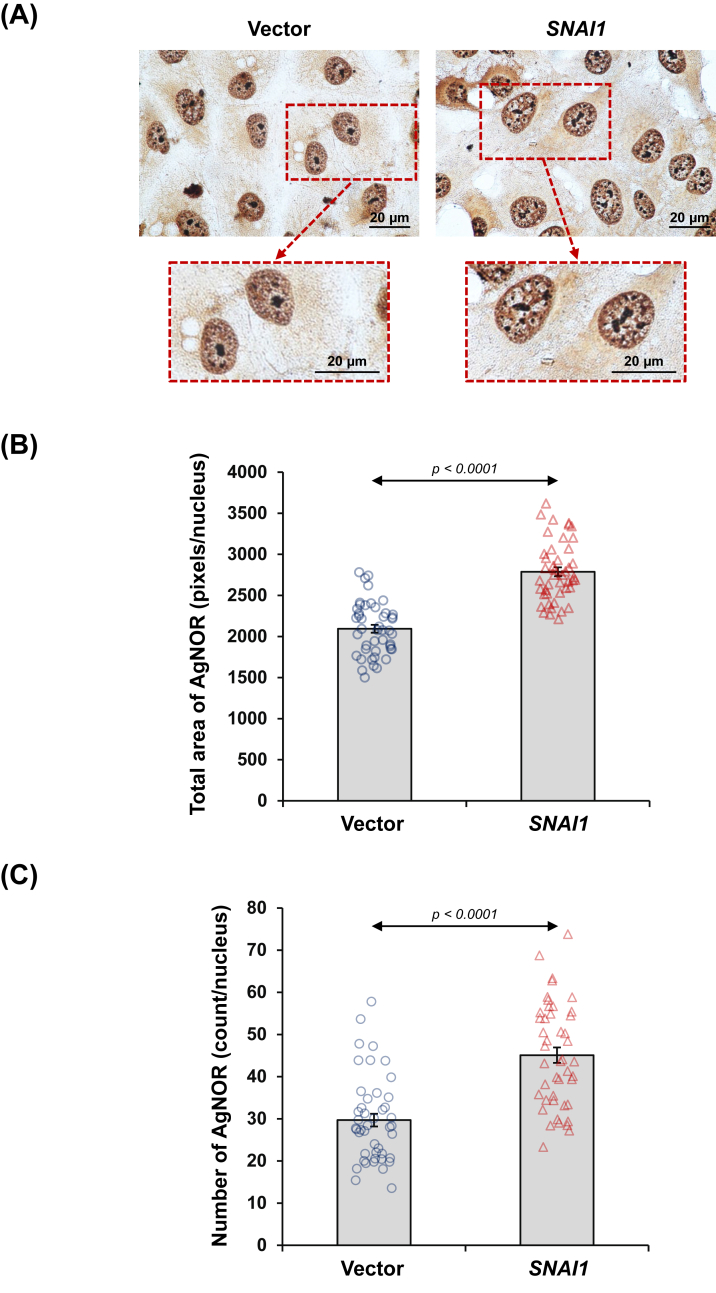
*SNAI1* overexpression increased nucleolar organizer regions (NORs) in renal tubular cells. (**A**) The NORs in vector-control and *SNAI1*-overexpressed HK-2 cells were evaluated by AgNOR staining. The inset indicates the zoom-in image of the highlighted area. **(B, C)** The NORs were quantified in individual nuclei from at least 15 random fields per sample based on (B) their total area per nucleus and (C) their number per nucleus. Each bar shows the mean ± standard error of the mean derived from three independent experiments.

According to KEGG pathway analysis, the enriched ribosome pathway drew our attention to experimental validation of ribosome biogenesis. Expression and localization of nucleophosmin, a nucleolar protein essential for the assembly and maturation of ribosomes, were examined via immunofluorescence assay. Nucleophosmin was intensified in the nucleoli of *SNAI1*-overexpressed cells when compared with the vector-control cells (Fig. 6A and B). Additionally, we determined the nucleolar organizer regions (NORs), the sites on chromosomes housing genes responsible for ribosomal RNA (rRNA) synthesis. The AgNOR staining revealed a remarkable increase in the total area of NORs as well as the number of NORs inside the nuclei of *SNAI1*-overexpressed cells (Fig. 7A–C).

### *SNAI1* overexpression induced cell enlargement and increased the granularity of renal tubular cells

**Figure 8:**
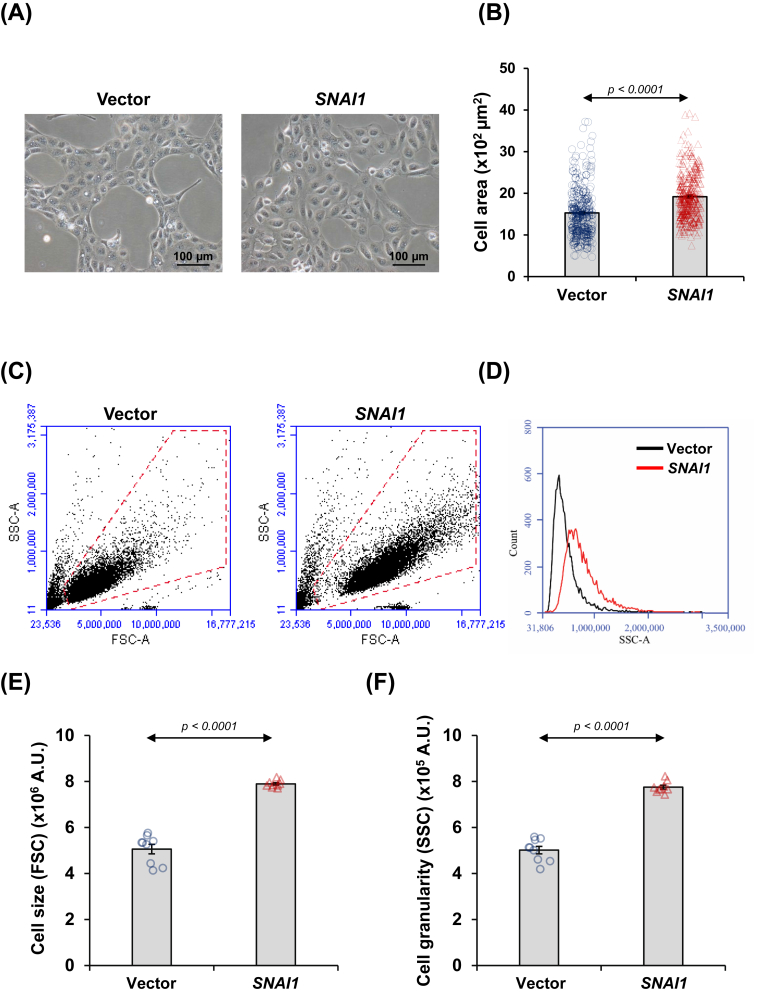
*SNAI1* overexpression induced cell enlargement and increased granularity of renal tubular cells. **(A)** The vector-control and *SNAI1*-overexpressed HK-2 cells were imaged under a phase contrast microscope. **(B)** Cell areas were measured from at least 100 cells in 10 random fields per sample. **(C)** The cells were also analyzed by flow cytometry. Representative scatter plots show forward scatter (FSC; indicating cell size) on the *x*-axis and side scatter (SSC; indicating cell granularity) on the *y*-axis. **(D)** The overlaid histogram plot displays a shift in cell granularity (SSC) of *SNAI1*-overexpressed HK-2 cells compared with vector-control cells. **(E, F)** Cell size (E) and cell granularity (F) were quantified and averaged from at least 10,000 cells per sample. Each bar shows the mean ± standard error of the mean derived from three independent experiments.

Our next step was to examine whether the ectopic *SNAI1* expression could induce renal tubular cell senescence. Microscopic observations showed a distinct difference in cell area between vector-control and *SNAI1*-overexpressed cells, notably showing the increased cell area in the *SNAI1*-overexpressed cells (Fig. 8A and B). Flow cytometry affirmed the significant cell enlargement, correlating with increased forward scatter (FSC) (Fig. 8C and E). Moreover, *SNAI1*-overexpressed cells showed elevated side scatter (SSC), indicating an increase in internal complexity or intracellular granularity (Fig. 8D and F).

### *SNAI1* overexpression increased senescence markers in renal tubular cells

**Figure 9:**
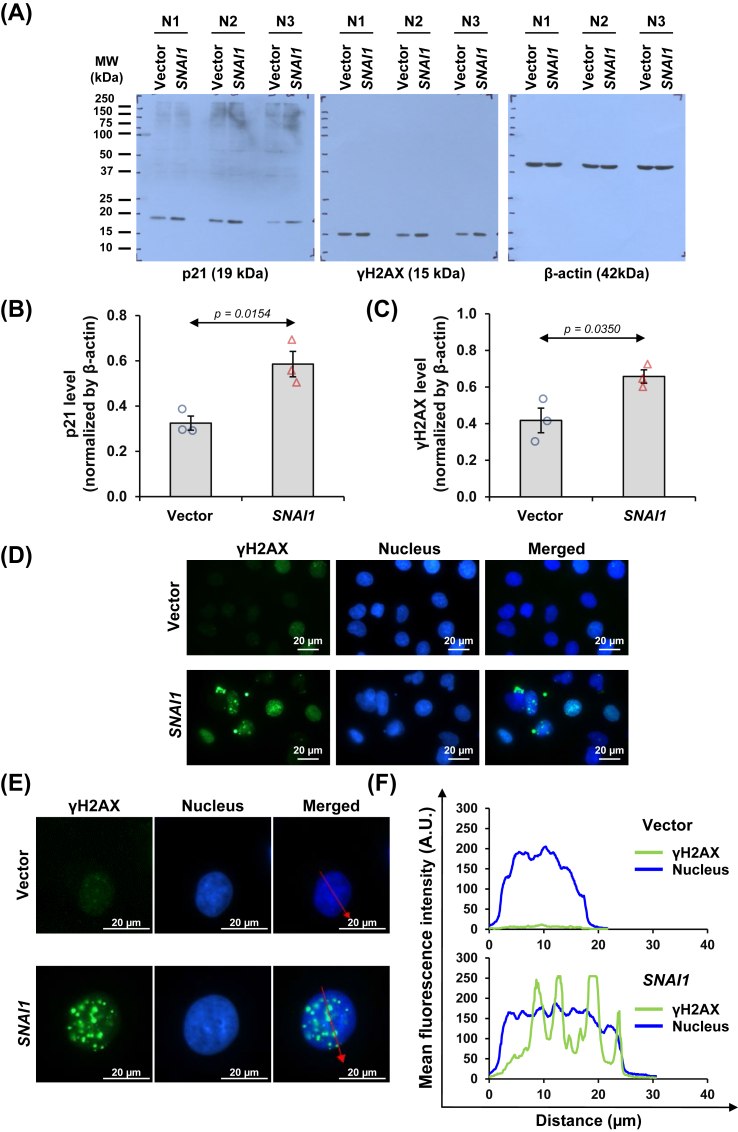
*SNAI1* overexpression increased senescence markers in renal tubular cells. **(A)** Levels of senescence markers, including p21 and γH2AX, in vector-control and *SNAI1*-overexpressed HK-2 cells were evaluated by immunoblotting. **(B, C)** Their band intensities were quantified and normalized by that of β-actin (loading control). Each bar shows the mean ± standard error of the mean derived from three independent experiments. **(D)** Expression of γH2AX was also evaluated by immunofluorescence assay. **(E)** Magnified images revealed the formation of γH2AX-foci within the nuclei as depicted by vivid green spots. The red line indicates the direction for fluorescence intensity spectral profiling. **(F)** The fluorescence intensity spectral profiles of γH2AX expression (illustrated by the green line) and nuclei (illustrated by the blue line).

The cell enlargement and increased granularity pointed toward characteristics related to cellular senescence. The senescence protein markers p21 and phosphorylated histone H2AX (γH2AX) were thus analyzed by immunoblotting, which revealed their significant increases in the *SNAI1*-overexpressed cells (Fig. 9A–C). The accumulation of γH2AX would lead to the formation of distinct γH2AX foci, which could be visualized as punctate structures (bright green spots) throughout the nuclei of *SNAI1*-overexpressed cells (Fig. 9D and E). This observation corresponded to the discrete fluorescence intensity spectral profile of γH2AX expression (displayed as the green spectrum), distinctly observed in the *SNAI1*-overexpressed cells (red arrows indicate the direction of the fluorescence intensity profile) (Fig. 9F). Hoechst intensity spectral profile of nuclei (shown as the blue spectrum) was continuous and comparable in both cells (Fig. 9F).

### *SNAI1* overexpression increased the production of MMPs from renal tubular cells

**Figure 10:**
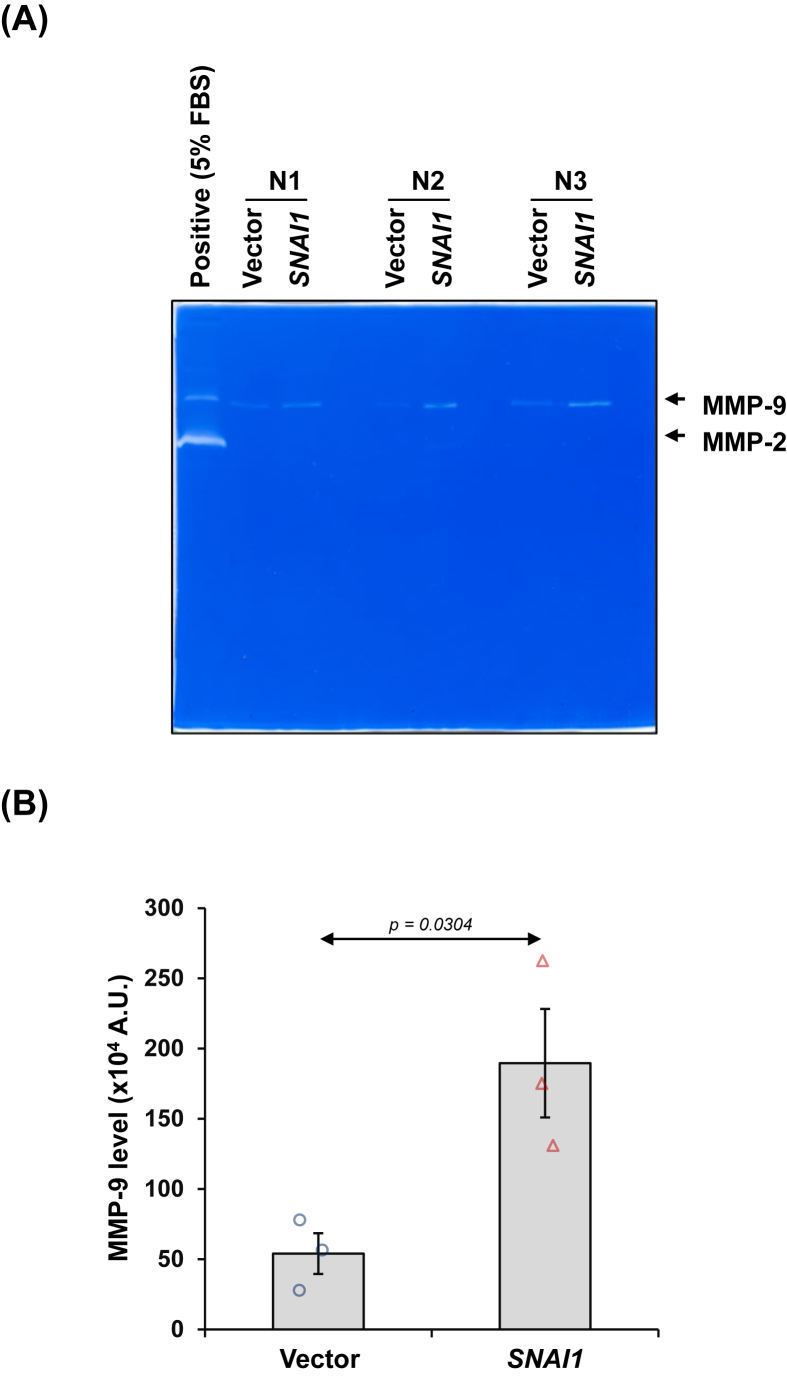
*SNAI1* overexpression increased the production of matrix metalloproteinase (MMP) from renal tubular cells. **(A)** MMP-9 in culture supernatants derived from vector-control and *SNAI1*-overexpressed HK-2 cells was evaluated by gelatin zymography. **(B)** MMP-9 band intensity was quantified. Each bar shows the mean ± standard error of the mean derived from three independent experiments.

MMP secretion, a key characteristic of the senescence-associated secretory phenotypes (SASPs), was assessed in the culture supernatant derived from vector-control and *SNAI1*-overexpressed cells. Gelatin zymography demonstrated a significant elevation of MMP-9 in *SNAI1*-overexpressed cells relative to vector-control cells (Fig. 10A and B), implicating its involvement in ECM remodeling.

### *SNAI1* overexpression induced focal adhesion organization in renal tubular cells

**Figure 11:**
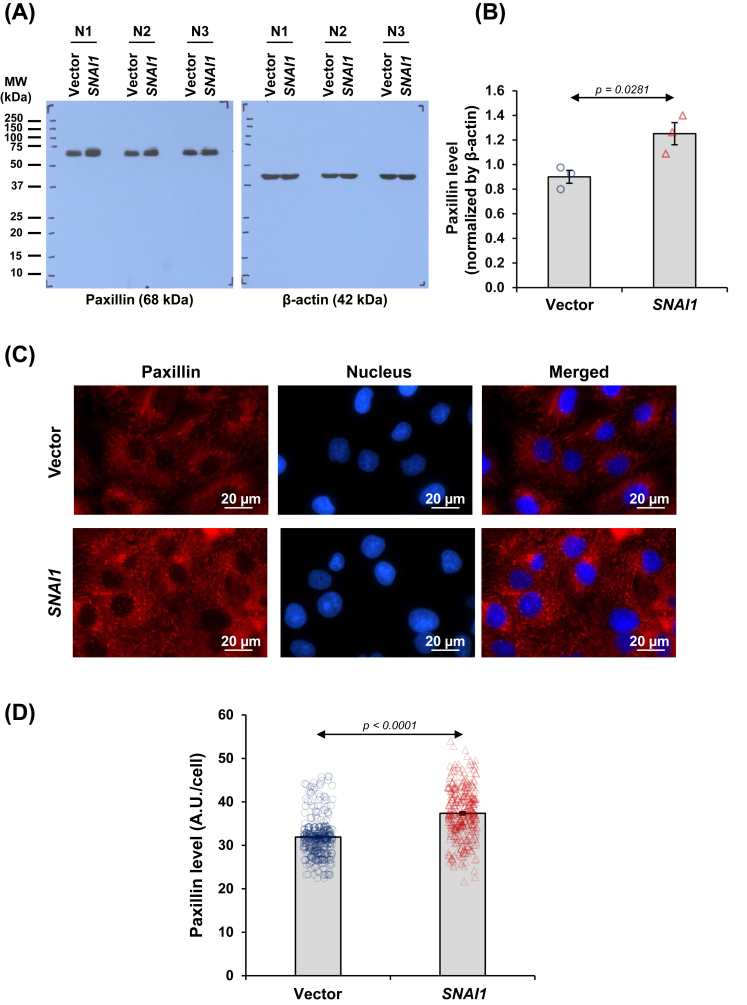
*SNAI1* overexpression induced focal adhesion organization in renal tubular cells. **(A)** Paxillin expression was evaluated in vector-control and *SNAI1*-overexpressed HK-2 cells by immunoblotting. **(B)** The paxillin band intensity was quantified and normalized by that of β-actin (loading control). **(C)** Expression and localization of paxillin were also examined by immunofluorescence assay. **(D)** The paxillin fluorescence intensity was evaluated in at least 100 cells across 10 random fields per sample. Each bar shows the mean ± standard error of the mean derived from three independent experiments.

According to the enrichment analysis of cellular components, focal adhesion was overrepresented by *SNAI1* overexpression. Immunoblot analysis revealed the notably up-regulated level of paxillin, the scaffolding protein localized at the focal adhesion foci, in *SNAI1*-overexpressed cells (Fig. 11A and B). In addition, the expression and organization of paxillin at focal adhesion foci were investigated by immunofluorescence assay. Consistently, the results illustrated that paxillin was up-regulated at the focal adhesion complex of *SNAI1*-overexpressed cells (Fig. 11C and D).

## Discussion

Snail1 is a crucial transcription regulator that controls the plasticity of renal epithelial cells upon adaptive response and injury, known as EMT.^25^ Targeted therapies aimed at this process may offer promising avenues for managing kidney fibrosis. In this study, we established stably *SNAI1*-overexpressed renal tubular cells and investigated alterations in the cellular proteome induced by the ectopic expression of *SNAI1*, which encodes the Snail1 protein. Immunoblotting of some altered proteins, such as the down-regulated HSP60 and HSP70, and the up-regulated DDX1, highly confirmed our LFQ proteomics data. By integrating LFQ proteomics and functional enrichment data, we pinpointed multiple biological processes significantly influenced by the ectopic *SNAI1* expression, involving the regulation of translation, ribosome, cell cycle, and cellular senescence. Additionally, focal adhesion was overrepresented among other subcellular compartments impacted by *SNAI1* overexpression.

Our LFQ proteomics data highlighted the up-regulated expression of DDX1 (also known as DEAD-box helicase 1), a protein of the DEAD-box family of RNA helicases participating in RNA metabolic activities. It has recently been identified as one of the core components of the human tRNA ligase complex.^26^ The DDX1 activity is central to various cellular functions, including RNA splicing, translation initiation, RNA degradation, and anti-oxidative defense mechanism.^27^ Its role in genome stability has been uncovered in γ irradiation of HeLa cells, demonstrating an increase in DDX1 nuclear level and its colocalization with γH2AX and suggesting its role in DNA damage response.^28^ DNA hybrids are essential for ATM (ataxia telangiectasia mutated) signaling to mediate DDX1-γH2AX interaction.^28^ In the unilateral ureteral obstruction murine model of kidney fibrosis, the circular RNA circInpp5b is mechanically bound to DDX1 to enhance its lysosomal degradation, resulting in the mitigation of renal interstitial fibrosis.^29^ Additionally, DDX1 loss leads to defects in rRNA processing and activation of the ribosome stress-p53 pathway in mouse embryonic stem cells.^30^ The increased DDX1 in *SNAI1*-overexpressed cells was thus indicative of the DNA damage response as well as the aberrant ribosome biogenesis, which is associated with the EMT phenotype.

Accordingly, the overrepresented ribosome by KEGG pathway analysis was concomitant with the increased DDX1 in *SNAI1*-overexpressed cells. These findings guided us to investigate ribosome biogenesis, the canonical process crucial for protein synthesis and cellular functions.^31^ This process primarily occurs in the nucleolus, with additional steps in the nucleoplasm and cytoplasm. Besides ribosome biogenesis, the nucleolus hosts a wide range of proteins involved in several biological processes, including ribonucleoprotein assembly, stress response, and cell cycle regulation.^32^^,^^33^ Thus, abnormalities in the function and architecture of this nuclear compartment devoted to ribosome biogenesis can trigger nucleolar stress, a mechanism connected to cellular aging.^34^ Additionally, the DNA damage response and oncogenic stress can trigger nucleolar stress responses.^34^ In our study, nucleophosmin, the nucleolar protein, was up-regulated in *SNAI1*-overexpressed cells. Apart from its primary role in ribosome biogenesis, nucleophosmin participates in regulating nucleolar stress by modulating interactions with diverse stress-related proteins and influencing cell cycle progression and DNA damage response.^33^^,^^35^

Moreover, we noted a significant increase in AgNORs in terms of their total area and number in *SNAI1*-overexpressed cells. Nucleolar organizer regions (NORs) are specific chromosomal regions harboring rRNA genes essential for ribosome biogenesis through organizing rRNA synthesis and processing.^36^ An increase in NORs directly correlates with nucleolar size and ribosome biogenesis. Dysregulated ribosome biogenesis is a hallmark of cancerous cells, supporting tumor progression by adapting to altered translational demands.^37^ In breast cancer, the recruitment of Snail1, RNA polymerase I, and upstream binding factor to the nucleolus is related to the increased ribosome biogenesis and EMT features.^37^ Additionally, in response to ribosome stress, Snail1 can trigger the JNK-USP36 signaling pathway, leading to Snail1 stabilization in the nucleolus that, in turn, enhances ribosome biogenesis and the survival of solid tumors.^38^

Interestingly, several lines of evidence have reported that a decrease in ribosome biogenesis and protein translation can extend the lifespan of organisms by reducing cellular energy expenditure.^39^, ^40^, ^41^, ^42^ Specifically, the longer lifespan is linked to smaller nucleoli.^39^^,^^42^ A study in *Caenorhabditis elegans* has demonstrated that longevity-related pathways converge within the nucleolus, and a decrease in ribosome biogenesis is associated with extended lifespan.^39^ Consistent findings are also evident in dietary-limited fruit flies, insulin-like-peptide mutant flies, *Irs1*-knockout mice with dietary restriction, and muscle biopsies among elderly individuals with restricted caloric intake and increased physical activity.^39^ Conversely, fibroblasts derived from patients with Hutchinson–Gilford progeria syndrome (premature aging disorder) exhibit increased nucleolar size and ribosome biogenesis, suggesting a connection between elevated ribosomal activity and premature senescence.^43^ The overall evidence underscores smaller nucleoli as a conserved cellular marker of longevity and metabolic health across multiple species. Taken together, we hypothesized that the ectopic *SNAI1* expression could introduce an imbalance in ribosome biogenesis by altering the expression level of nucleolar components (*i.e.*, nucleophosmin, nucleolin, and ribosomal proteins (see details in Table 1)), increasing nucleolar size, and triggering DNA damage response, nucleolar stress, and cellular senescence in *SNAI1*-overexpressed cells.

In line with this hypothesis, KEGG pathway analysis emphasized the key role of cellular senescence. We thus sought to investigate the phenotypic changes related to senescence in *SNAI1*-overexpressed cells. In kidney diseases, proximal tubular cells are commonly found as the major cell type undergoing senescence.^44^^,^^45^ Senescent cells encounter changes in both cell morphology and epigenetics, *e.g.*, cell enlargement, increased granularity, cellular flattening, altered shape, intensified nuclear staining, chromatin aggregation, defective DNA replication, increased expression of β-galactosidase 1 activity, and apoptosis resistance.^45^, ^46^, ^47^ Senescent cells display irreversible growth arrest accompanied by up-regulation of key cyclin-dependent kinase inhibitors, including p16INK4a (p16) and p21CIP1 (p21). Additionally, senescent cells show signs of persistent DNA damage (such as unresolved γH2AX foci) and impaired DNA repair mechanisms.^48^^,^^49^ According to the described characteristics, we noted the increased cell size and granularity of *SNAI1*-overexpressed cells. This observation aligned with the previous findings on H_2_O_2_-induced senescence in HK-2 cells.^50^ The cell enlargement and increased granularity arise from several cytoplasmic and cellular alterations, *e.g.*, the enlarged organelle size and accumulation of lysosomes and cellular materials due to the inefficient clearance of cellular debris and macromolecules.^51^ These factors collectively contribute to the distinctive enlargement and irregular shape of senescent cells, reflecting the profound changes associated with senescence.

In addition, *SNAI1*-overexpressed cells had up-regulated expression of senescence markers, including p21 and γH2AX, the phosphorylated histone variant H2AX (at serine 139). Beyond its critical role in cellular responses to DNA damage and repair, γH2AX is associated with cellular senescence.^52^ Following a DNA double-strand break, γH2AX swiftly accumulates at the break site, resulting in the formation of discrete foci that serve as the platforms to recruit the proteins involved in DNA repair.^52^ In the context of kidney aging, γH2AX foci formation has been reported in senescent podocytes of streptozotocin-induced diabetic mice that can be mitigated by a ketone body, β-hydroxybutyrate, via reducing glycogen synthase kinase-3 beta (GSK3β) activity and activating the nuclear factor erythroid 2-related factor 2 (Nrf2) pathway.^53^ Interestingly, overexpression of Klotho, an anti-aging protein, has demonstrated a renoprotective effect on human renal cells and mouse renal tissue exposed to radiation, as evidenced by improved cell survival and reduced γ-H2AX foci formation, highlighting its role against chromosomal DNA damage.^54^ Our findings demonstrated a marked elevation in foci formation in *SNAI1*-overexpressed cells, pointing to the activation of DNA damage response triggered by genotoxic stress induced by ectopic *SNAI1* expression, which localized predominantly in the nucleus.

Importantly, a hallmark of senescence is the senescence-associated secretory phenotypes (SASPs), in which senescent cells release various chemokines, growth factors, pro-inflammatory cytokines, and ECM-degrading enzymes, contributing to tissue inflammation, ECM remodeling, and fibrosis.^55^^,^^56^ Certain MMPs like MMP-1, MMP-2, MMP-7, MMP-9, and MMP-13 have been implicated in the aging kidney.^57^, ^58^, ^59^, ^60^, ^61^ Specifically in CKD, MMP-2, MMP-7, MMP-9, and MMP-14 contribute to ECM deposition in the glomeruli and initiate cellular junction shedding and EMT in renal tubules, with MMP-2 and MMP-9 notably linked to systemic vascular damage through the increased TGF-β.^57^, ^58^, ^59^, ^60^, ^61^ A recent investigation has revealed that elevations of MMP-9 and growth differentiation factor-15 (GDF-15) specifically link to diabetes in females but not in males.^62^ Intriguingly, MMP-9 plays significant roles in multiple signaling pathways, including p38, Notch, and nuclear factor kappa B (NF-κB), to intensify inflammation, and thus serves as a promising therapeutic target for kidney fibrosis.^63^ Our results by gelatin zymography displayed a substantial increase only in MMP-9 production, but not that of MMP-2, in the cultured supernatant of *SNAI1*-overexpressed cells, indicating enhanced proteolytic activity to degrade ECM components and a series of intracellular signaling.

Furthermore, unraveling the complexity of cell-ECM dynamics may foster the identification of new targets for treating kidney fibrosis. In this study, the results of enrichment analysis of cellular components revealed that focal adhesion was overrepresented. Focal adhesion is a specialized structure, where cells maintain their interior-ECM interactions and mediate signaling to influence gene expression and cytoskeletal dynamics.^64^^,^^65^ A recent study has shown that hyperosmotic stress can drive EMT through the reorganization of focal adhesions and actin filaments in renal tubular cells.^66^ The process can be counteracted by a selective Rho-associated protein kinase inhibitor, Y-27632 ^66^. Analysis of renal allograft biopsies has revealed a link between EMT and progressive interstitial fibrosis, proteinuria, and graft survival.^67^ Co-expression of mesenchymal markers, such as vinculin, paxillin, and alpha-smooth muscle actin (α-SMA), in tubular cells and glomerular podocytes has a direct correlation with the severity of interstitial fibrosis and proteinuria, respectively.^67^ According to the enrichment results, we observed a notably elevated level of paxillin, a scaffold protein found in focal adhesions, in *SNAI1*-overexpressed cells. This finding is consistent with the increased paxillin and its phosphorylated form observed in TGF-β2-mediated EMT of retinal pigment epithelial cells.^68^^,^^69^

Paxillin is pivotal for transmitting signals bidirectionally across cell membranes, relaying outside-in signaling from the ECM into the cell interior that influences cell migration, proliferation, and survival.^70^ Importantly, paxillin plays dual roles in focal adhesion dynamics by regulating nascent formation (transient adhesive structure) and maturation, which is essential for cellular response to temporal environmental changes or during cell migration.^71^ In this study, besides substantially increased paxillin levels, we also observed elongated paxillin in vector-control cells and its short form in *SNAI1*-overexpressed cells, indicating the presence of mature and nascent focal adhesions, respectively. The investigation of mechanosensing organelles in eukaryotic cells has revealed the importance of the formation of nascent focal adhesion in cell spreading and differentiation on substrates.^72^ It is conceivable that a significant increase in nascent focal adhesion driven by ectopic *SNAI1* expression could affect cell adhesion and promote cell migration.^71^ This finding may be attributed to the dynamic turnover of mature focal adhesion, which is vital for cell migration and remodeling processes through the interaction of paxillin with regulatory proteins, such as focal adhesion kinase (FAK), Src, vinculin, and other signaling molecules.^71^ Furthermore, a recent study has highlighted FAK-dependent intratubular EMT as a crucial mechanism driving tubular atrophy after severe kidney injury.^73^ Targeting this pathway may potentially alleviate tubular atrophy and interstitial fibrosis during a chronic phase.^73^ Thus, the increased expression of paxillin implies that the ectopic *SNAI1* expression may trigger the FAK pathway. Beyond the structural and signaling functions, mature focal adhesion can influence cell shape, polarity, and cytoskeletal organization. This might also account for alterations in cell shape observed in *SNAI1*-overexpressed cells, in which mature focal adhesion was scarcely observed.

## Conclusions

*SNAI1*-overexpressed cells displayed cellular proteome alterations and increases in nucleophosmin, NORs, cell size, granularity, p21, γH2AX, MMP-9 secretion, and paxillin expression. This study has broadened our knowledge of Snail1 functions beyond its established role as the EMT regulator. In addition to alterations in the cellular proteome, ectopic *SNAI1* expression induced nucleolar stress, ribosome biogenesis, senescence, and DNA damage response in renal tubular cells. Moreover, Snail1 also affected the dynamics of focal adhesion, which is imperative for cell migration, by regulating paxillin expression. Our findings may offer new therapeutic targets related to Snail1-dependent mechanisms for the effective management of kidney fibrosis.

## CRediT authorship contribution statement

**Rattiyaporn Kanlaya:** Writing – review & editing, Writing – original draft, Visualization, Validation, Methodology, Investigation, Funding acquisition, Formal analysis, Data curation, Conceptualization. **Kanokwan Nonthawong:** Writing – review & editing, Validation, Methodology, Investigation, Formal analysis, Data curation, Conceptualization. **Mueanchan Suntivichaya:** Writing – review & editing, Validation, Methodology, Investigation, Formal analysis, Data curation, Conceptualization. **Sunisa Yoodee:** Writing – review & editing, Validation, Methodology, Investigation, Formal analysis, Data curation, Conceptualization. **Visith Thongboonkerd:** Writing – review & editing, Writing – original draft, Validation, Supervision, Software, Resources, Project administration, Methodology, Investigation, Conceptualization.

## Data availability

The mass spectrometry proteomics data have been deposited to the ProteomeXchange Consortium (http://www.proteomexchange.org/) via the PRIDE (https://www.ebi.ac.uk/pride/) partner repository with the dataset identifier PXD061033 (Username: reviewer_pxd061033@ebi.ac.uk/Pass: 0TcuBMfLhvnO).

## Funding

This study was funded by the 10.13039/501100004704National Research Council of Thailand (NRCT) and 10.13039/501100004156Mahidol University (No. N42A650361).

## Conflict of interests

The authors declared no conflict of interests.
