# Supplementary material for: Deciphering the impact of SNAI1 gene on renal tubular cell proteome, nucleolar stress, ribosome biogenesis, senescence, DNA damage response, and focal adhesion dynamics
Source: Genes Dis. 2025 Nov 10;13(4):101926. doi: 10.1016/j.gendis.2025.101926 (PMC13091999; doi:10.1016/j.gendis.2025.101926)
Supplement: Multimedia component 1 [file mmc1.pdf]

SUPPORTING INFORMATION

Deciphering the impact of *SNAI1* gene on renal tubular cell proteome, nucleolar stress, ribosome biogenesis, senescence, DNA damage response, and focal adhesion dynamics

*Rattiyaporn Kanlaya, Kanokwan Nonthawong, Mueanchan Suntivichaya, Sunisa Yoodee, and Visith Thongboonkerd**
*Correspondence to: thongboonkerd@dr.com (or) vthongbo@yahoo.com

Table S1. Downstream members of the individual top 10 transcription factors triggered by *SNAI1* overexpression.

Rank	Transcription factor	Downstream members									
1	**HMGA1**	RPL4	EIF4A1	DAZAP1	RPL3	HSP90AB1	TFRC	PRKCSH	HSPB1	YBX1	RPL8
2	**E2F4**	RPL4	EIF4A1	RPL3	TFRC	HNRNPR	RPL8	PHB2	ACTG1	RPL7	PPP1CC
3	**TFDP1**	RPL4	DAZAP1	HSP90AB1	TFRC	PRKCSH	HNRNPR	YBX1	RPL8	PHB2	LMNB1
4	**MYC**	RPL4	EIF4A1	RPL3	TFRC	RPL34	PDCD5	STMN2	HNRNPR	RPL8	PHB2
5	**TP53**	EIF4A1	RPL3	TFRC	PLOD2	RPL8	PHB2	ACTG1	EEF1B2	RPS14	PPP1CC
6	**ZNF207**	RPL4	EIF4A1	RPL3	TFRC	HNRNPR	RPL8	PHB2	RPL6	ACTG1	RPS14
7	**ZNF581**	RPL4	RPL3	PRKCSH	RPL34	HSPB1	RPL8	PHB2	ACTG1	EEF1B2	RPS14
8	**PRMT3**	RPL4	EIF4A1	HSP90AB1	TFRC	PRKDC	DHX9	DDX1	GDI2	GMPS	PHB2
9	**DDIT3**	DAZAP1	TFRC	AHNAK	SHMT2	PRKCSH	DDX1	SUMO4	HSPB1	YBX1	HSP90B1
10	**NKRF**	RPL4	HSP90AB1	TFRC	YWHAB	STMN2	YBX1	LMNB1	RPL7A	RPS17	KHSRP
